# Molecular evidence of field cancerization initiated by diabetes in colon cancer patients

**DOI:** 10.1002/1878-0261.12438

**Published:** 2019-02-16

**Authors:** Laura Del Puerto‐Nevado, Pablo Minguez, Marta Corton, Sonia Solanes‐Casado, Isabel Prieto, Sebastian Mas, Ana Belen Sanz, Paula Gonzalez‐Alonso, Cristina Villaverde, Sergio Portal‐Nuñez, Oscar Aguilera, Carmen Gomez‐Guerrero, Pedro Esbrit, Fernando Vivanco, Nieves Gonzalez, Carmen Ayuso, Alberto Ortiz, Federico Rojo, Jesus Egido, Gloria Alvarez‐Llamas, Jesus Garcia‐Foncillas, the DiabetesCancerConnect Consortium

**Affiliations:** ^1^ Translational Oncology Division Oncohealth Institute IIS‐Fundacion Jimenez Diaz‐UAM Madrid Spain; ^2^ Genetics Department IIS‐Fundacion Jimenez Diaz‐UAM Madrid Spain; ^3^ Center for Biomedical Network Research on Rare Diseases (CIBERER) ISCIII Madrid Spain; ^4^ Radiation Oncology Oncohealth Institute IIS‐Fundacion Jimenez Diaz‐UAM Madrid Spain; ^5^ Renal, Vascular and Diabetes Research Laboratory IIS‐Fundacion Jimenez Diaz‐UAM Spanish Biomedical Research Network in Diabetes and Associated Metabolic Disorders (CIBERDEM) Madrid Spain; ^6^ Nephrology and Hypertension Department IIS‐Fundacion Jimenez Diaz‐UAM Madrid Spain; ^7^ REDINREN Madrid Spain; ^8^ Pathology Department IIS‐Fundacion Jimenez Diaz‐UAM Madrid Spain; ^9^ Bone and Mineral Metabolism Laboratory IIS‐Fundacion Jimenez Diaz‐UAM Madrid Spain; ^10^ Applied Molecular Medicine Institute School of Medicine Universidad San Pablo CEU CEU Universities Madrid Spain; ^11^ Immunoallergy and Proteomics Laboratory Immunology Department IIS‐Fundacion Jimenez Diaz‐UAM Madrid Spain

**Keywords:** carcinogenesis, colon cancer, diabetes, field cancerization, omics, systems biology

## Abstract

The potential involvement of type 2 diabetes mellitus (T2DM) as a risk factor for colon cancer (CC) has been previously reported. While several clinical studies show a higher incidence of CC and a lower survival rate in diabetics, others report no association. Our own experience indicates that diabetes does not seem to worsen the prognosis once the tumor is present. Despite this controversy, there are no wide‐spectrum molecular studies that delve into the impact of T2DM‐related mechanisms in colon carcinogenesis. Here, we present a transcriptomic and proteomic profiling of paired tumor and normal colon mucosa samples in a cohort of 42 CC patients, 23 of which have T2DM. We used gene set enrichment and network approaches to extract relevant pathways in diabetics, referenced them to current knowledge, and tested them using *in vitro* techniques. Through our transcriptomics approach, we identified an unexpected overlap of pathways overrepresented in diabetics compared to nondiabetics, in both tumor and normal mucosa, including diabetes‐related metabolic and signaling processes. Proteomic approaches highlighted several cancer‐related signaling routes in diabetics found only in normal mucosa, not in tumors. An integration of the transcriptome and proteome analyses suggested the deregulation of key pathways related to colon carcinogenesis which converged on tumor initiation axis TEAD/YAP‐TAZ as a potential initiator of the process. *In vitro* studies confirmed upregulation of this pathway in nontumor colon cells under high‐glucose conditions. In conclusion, T2DM associates with deregulation of cancer‐related processes in normal colon mucosa adjacent to tissue which has undergone a malignant transformation. These data support that in diabetic patients, the local microenvironment in normal colon mucosa may be a factor driving field cancerization promoting carcinogenesis. Our results set a new framework to study links between diabetes and colon cancer, including a new role of the TEAD/YAP‐TAZ complex as a potential driver.

**Abbreviations**
ADA: American Diabetic Association
BMI: body mass index
CC: colon cancer
CEIC: Institutional Scientific and Ethical Committee
FASP: filter‐aided sample preparation
FBS: fetal bovine serum
FC: fold change
FDR: false discovery rate
FFPE: formalin‐fixed paraffin‐embedded
FWHM: full width at half maximum
GEO: gene expression omnibus
GSE: gene set enrichment
iTRAQ: Isobaric Tags for Relative and Absolute Quantitation
KEGG: Kyoto Encyclopedia of Genes and Genomes
LOPD: organic law on protection of personal data
MCN: minimal connected network
MS/MS: tandem mass spectrometry
MSI: microsatellite‐instable
N: normal colonic mucosa from nondiabetic patients
ND: normal colonic mucosa from diabetic patients
PCA: principal component analysis
STZ‐D: streptozotocin‐induced diabetes
STZ: streptozotocin
T: tumor from nondiabetic patients
T2DM: type 2 diabetes mellitus
TD: tumor from diabetic patients
WHO: World Health Organization
WT: wild‐type

## 1 Introduction

The association between type 2 diabetes mellitus (T2DM) and colon cancer (CC) has been extensively discussed (González *et al*., 2017). While several cohort studies and meta‐analyses have reported an increased risk for CC development in T2DM patients (Larsson *et al*., 2005; Sun and Yu, 2012) as well as a higher short‐ and long‐term mortality (Croft *et al*., 2018; Zhu *et al*., 2017), others identified biases produced by the size and origin of the populations studied (de Jong *et al*., 2017; Tsilidis *et al*., 2015). Our own experience using a highly homogeneous Spanish cohort indicated that even if there is a higher risk of cancer development, the effect of diabetes does not entail a worse outcome once the tumor has developed (Prieto *et al*., 2017). In any case, the controversies generated by the different observational studies have strengthened the need to study the molecular mechanisms behind a possible association between both diseases, today still unclear. In this context, some authors have proposed several metabolic pathways, such as the insulin pathway or oxidative stress, as important players linking diabetes to promotion of tumor development or metastasis (Ikemura *et al*., 2013; Teng *et al*., 2016; Yang *et al*., 2017). However, there is very limited information derived from patient samples supporting these evidences.

The concept of field cancerization was proposed for the first time by Slaughter *et al*. and emerged as a new paradigm in carcinogenesis (Slaughter *et al*., 1953). This theory defines premalignant epithelial areas with normal histology that promote cancer development. Several factors have been proposed to drive oncogenesis from inflammatory diseases (Galandiuk *et al*., 2012; Leedham *et al*., 2009). In sporadic colon cancer, a field effect has been reported in the normal mucosa surrounding the tumor based on changes in methylation patterns (Shen *et al*., 2005), chromosomal instability, copy number alterations (Hawthorn *et al*., 2014), or even in Warburg metabolism (Cruz *et al*., 2017). T2DM has also been proposed as a factor for field cancerization in *in vitro* experiments (Rubin, 2013).

With all these evidences in hand, we designed a ‘proof‐of‐concept’ study to shed light on the impact of T2DM as a driver for the field cancerization in normal mucosa that could explain a higher risk of CC. To this end, we combined transcriptomic and proteomic approaches in a comprehensive, unbiased analysis of surgical biopsies from CC patients to extract the molecular profile of normal mucosa in both nondiabetic and T2DM patients. For the first time, we describe signaling pathways exclusively activated in diabetic normal mucosa which could contribute as triggering events for colon carcinogenesis. These findings were expanded with experimental studies in cultures of a normal mucosa cell line using different glucose concentrations. Altogether, we propose a novel molecular scenario where diabetes could promote a precancer state.

## 2 Materials and methods

### 2.1 Patient selection and study population

Patients were recruited from January 2009 to December 2013 at the Fundación Jiménez Díaz Hospital (Madrid, Spain). The same cohort was used in a previous epidemiological study (Prieto *et al*., 2017). The inclusion parameters were as follows: patients with resection of primary colon cancer, colon adenocarcinoma histological type, colon location (rectal cancer patients were excluded), time from surgery up to 6 months, no neoadjuvant treatment, no other concurrent neoplasia or immunosuppressive treatment, and diabetes diagnosed as a documented registry of diabetes, or historical antidiabetic medication intake or meeting the American Diabetic Association (ADA) criteria for diabetes at time of reviewing the data. The ADA criteria used to determine if patients had diabetes were as follows: hemoglobin A1c values ≥ 6.5%, or fasting blood glucose levels ≥125 mg·dL^−1^, with high fasting values recorded 2 or more times, or random blood glucose levels ≥200 mg·dL^−1^, with high random values recorded two or more times. In parallel, this observational study also included 79 nondiabetic patients with primary diagnosis of colon cancer, who underwent resection during the same period, using equal inclusion criteria except the presence of diabetes, aiming to obtain a well‐balanced series. No significant differences in body mass index (BMI) were observed between diabetics and nondiabetics (Prieto *et al*., 2017).

A total of 160 patients met the inclusion criteria described above. From them, we made a subselection based on sample availability and with the purpose of obtaining a homogeneous and well‐balanced subset of patients. Thus, the final set of patients were selected in terms of tumor and clinical characteristics (gender, grade (low grade: G1–G2; and high grade: G3, following the 2010 WHO classification), tumor site (right: cecum, hepatic flexure, ascending and transverse colon; and left: splenic flexure and descending colon), stage (low stage: 0, I, any II; or high stage: any III, IV), recurrence, final status, and metformin intake). The final cohort composed of 42 patients (23 diabetic and 19 nondiabetic) was used to conduct this study with samples derived from colon cancer resection. The study was approved by the Institutional Scientific and Ethical Committee at IIS‐Fundación Jiménez Díaz (Madrid, Spain) (CEIC‐FJD, approval code 08/13; on October 1, 2013) in accordance with the ethical principles stated in the Declaration of Helsinki. Informed consent is included in the clinical history of each participant and recorded by the standard requirements of data protection rules established by the SPANISH DATA PROTECTION AGENCY (LOPD 15/1999).

### 2.2 Tissue sampling

Surgical resection specimens from colon cancer tumors were obtained from Fundacion Jimenez Diaz Biobank. Paired FFPE samples from tumor and nontumor adjacent normal colonic mucosa from each individual were selected. Cancer tissue was obtained from the resected tumor edge, and the percentage of tumor content in FFPE samples was more than the 70%. Normal colonic mucosa samples were selected from a > 5 cm distance from the tumor. Pathologists confirmed the absence of morphological lesions in the normal colonic tissue.

### 2.3 Proliferation, MSI phenotype, and RAS and BRAF mutational analysis of samples

Mutational analysis for BRAF, KRAS, and NRAS genes was performed on FFPE CCR samples by pyrosequencing and PCR‐based assay. Briefly, DNA was isolated from 20 μm of representative tumor tissue. KRAS and NRAS were studied by pyrosequencing using the therascreen KRAS and RAS Extension Pyro Kits (Qiagen, Venlo, The Netherlands), following the manufacturer's recommendations. BRAF was assayed by the PCR‐based Cobas 4800 BRAF V600 Mutation Test (Roche, Basel, Switzerland).

The MSI phenotype was studied by testing the expression of the four MMR proteins (MLH1, MSH2, MSH6, and PMS2) by immunohistochemistry on Omnis platform (Dako, Glostrup, Denmark), using conventional 3‐μm tissue sections from the same specimens. Interpretation of staining was performed by qualified pathologists. Finally, proliferation was estimated as percentage by labeling Ki67 expression in tumor cells by immunohistochemistry on Omnis platform.

### 2.4 Xenograft model

Tumor xenografts in mice with streptozotocin‐induced diabetes were developed using methods extensively described in our previous report (Prieto *et al*., 2017). Briefly, 8‐week‐old athymic mice NU‐Foxn1nu (15 mice) (Charles River Laboratories, Wilmington, MA, USA) were injected with 200 mg·kg^−1^ body weight streptozotocin (STZ, Sigma‐Aldrich, Darmstadt, Germany) to achieve a diabetic environment. The control group (five mice) received vehicle. Ten days after STZ administration, 60% of STZ‐injected animals presented blood glucose above 200 mg·dL^−1^ and were considered as the streptozotocin‐induced diabetes (STZ‐D) group.

The colorectal cancer HT29 cell line was used to generate a xenograft model twenty days after STZ or vehicle administration. 2 × 10^6^ cells were injected subcutaneously into both flanks of the animals, and tumor growth was monitored. Fifty‐five days after tumor induction, mice were sacrificed, and tumors were removed and frozen for microarray study.

All animal procedures were approved by the Ethical Animal Research Committee at IIS‐Fundación Jiménez Díaz (Madrid, Spain) and were also conducted in accordance with institutional standards (reference number: PROEX 024‐15), which fulfilled the requirements established by the Spanish government and the European Community (Real Decreto R.D. 53/2003).

### 2.5 Gene expression analysis by GeneChip arrays

Total RNA from each human surgical sample was extracted from FFPE (5 μm thick) using RNeasy FFPE Kit (Qiagen) following the manufacturer's instructions. RNA derived from xenograft experiments was extracted with RNeasy Kit (Qiagen). In both microarray experiments, RNA was quantified using Qubit Fluorometric Quantitation (ThermoFisher, Waltham, MA, USA) and samples were processed using Affymetrix GeneChip^®^ Human Gene 2.0 ST Array. Labeling and hybridizations were performed according to Affymetrix protocols. Briefly, 50 ng total RNA was amplified and labeled using the WT Pico Reagent Kit (Affymetrix, Santa Clara, CA, USA) and then hybridized to Human Gene 2.0 ST Array (Affymetrix). Washing and scanning were performed using GeneChip System of Affymetrix (GeneChip Hybridization Oven 645, GeneChip Fluidics Station 450, and GeneChip Scanner 7G).

Microarray CEL files from human and xenograft samples were separately managed, corrected for background, and normalized using the RMA method implemented in the oligo r package (Carvalho and Irizarry, 2010) and annotated with hugene20sttranscriptcluster r package. PCA was performed with *pca3d* R library. The Babelomics suite was used for merging replicates and assessing differential expression using *limma* implementation with FDR p‐value correction. Data from the human and xenograft microarrays have been deposited in NCBI's Gene Expression Omnibus (GEO) with accession numbers GSE115313 and GSE115329, respectively.

### 2.6 Differential protein analysis by (iTRAQ)‐LC‐MS/MS

Ten slices 5 μm thick were collected from each FFPE sample. Four biological replicates were analyzed per condition (T, N, TD, and ND), combining 8 samples (T or N) and 6 samples (TD or ND) per replicate in the case of nondiabetic and diabetic patients, respectively.

Tissue was deparaffinized and proteins extracted as previously described (Gámez‐Pozo *et al*., 2012). Total protein was quantified by the BCA Protein Assay Kit (Thermo Scientific). Four biological replicates were analyzed per condition (T, N, TD, and ND), combining 8 samples (T or N) and 6 samples (TD or ND) per replicate in the case of nondiabetic and diabetic patients, respectively. Digestion was performed using the filter‐aided sample preparation (FASP) method (Wiśniewski *et al*., 2009). Briefly, proteins were reduced in 15 mm TCEP and alkylated in 50 mm of IAA and samples were cleaned five times with 8 m urea and 0.1 m TEAB (UTEAB). The first digestion using endoproteinase Lys‐C (1 : 100 w/w; Wako Pure Chemical Industries, Osaka, Japan) was performed overnight at room temperature in a wet chamber, followed by a dilution ninefold in 100 mm TEAB to reduce urea concentration. The second digestion using trypsin (1 : 100 w/w; Promega, Madison, WI, USA) was performed during 4 h at 37 °C in agitation (300 rpm). Digestions were stopped by the addition of TFA, and 75 μg of each tryptic digest was labeled according to the manufacturer's instructions (AB Sciex, Darmstadt, Germany) with one 8‐plex isobaric amine‐reactive tag per cell line (iTRAQ^®^ Reagent 8plex kit). Labeled samples were combined, cleaned up using a Sep‐Pak C18 cartridge for SPE (Waters Corp., Milford, MA, USA), and fractionated using the high‐pH reversed‐phase technique (Wang *et al*., 2011). All samples were analyzed by LC‐MS/MS on the LTQ Orbitrap Velos mass spectrometer (Thermo Scientific) coupled to an Eksigent nanoLC system (Eksigent, Darmstadt, Germany) through a nanoelectrospray ion source (Proxeon Biosystems). Peptides were loaded onto a ReproSil‐Pur C18‐Aq 5 μm 0.3 × 10 mm trapping cartridge (SGE Analytical) and washed for 10 min. The peptides were eluted from a RP ReproSil‐Pur C18‐AQ 2.4 μm 500 x 0.075 mm (Dr. Maisch GmbH, Ammerbuch‐Entringen) by a binary gradient consisting of 4% ACN in 0.1% FA (buffer A) and 100% ACN in 0.1% FA (buffer B), with a flow rate of 250 nL·min^−1^, as follows: 0–2 min 6% B, 2–133 min 30% B, and 133–143 min 98% B. The LTQ Orbitrap Velos was operated in positive ionization mode. The resolution was set to 30000 FWHM at m/z 400. The m/z values triggering MS/MS with a repeat count of 1 were put on an exclusion list for 40 s. The minimum MS signal for triggering MS/MS was set to 5000 counts. In all cases, one microscan was recorded. Higher‐energy dissociation (HCD) was used for fragmentation, up to the 15 most abundant isotope patterns with charge ≥2 from the survey scan were selected for fragmentation in the HCD collision cell. Normalized collision energy was set to 36.0 and activation time to 0.10 ms. Waveform filter was activated. The resulting fragments were detected in the Orbitrap system with a resolution of 7500 FWHM at m/z 400. The maximum ion injection times for the survey scan and the MS/MS scans were 500 ms and 250 ms, respectively, and the ion target values were set to 1E6 and 3E4, respectively, for each scan mode. Data files were analyzed using Proteome Discoverer 1.4 (Thermo Scientific) with Sequest HT as the search engine against a concatenated UniProt database of Homo sapiens (20,187 sequences) supplemented with frequently observed contaminants (397 sequences). iTRAQ 8plex tags in lysine and N terminus were included as fixed modifications, together with carbamidomethylation of cysteine. Oxidation of methionine was included as variable modification. Precursor mass tolerance was 20 ppm for all instruments, and fragment mass tolerance was 0.025. The integration of reporter ions was performed using the most confident centroid with a tolerance of 20 ppm. Reagents’ impurities were corrected as indicated by the manufacturer. PSMs were filtered using Percolator with a FDR of 1%. Quantification results at the PSM level were exported for further analysis.

Quantification and statistical analysis were performed using Isobar in R. We used a noise model that accounts for the technical variation due to the instrument. A null protein distribution was used to model sample variability (created by comparing biological replicates). Afterward, protein ratios were calculated for all the possible combinations and only proteins having both ‘*P*‐value sample’ and ‘*P*‐value ratio’ under 5% were considered significant.

### 2.7 Functional enrichment analyses

The functional enrichment analyses of transcriptomics and proteomics data were performed using the Babelomics suite of tools (Alonso *et al*., 2015) and the Kyoto Encyclopedia of Genes and Genomes (KEGG) as the source of annotation (Kanehisa *et al*., 2017). We performed gene set enrichment (GSE) analysis using logistic model module for the microarray data (Montaner and Dopazo, 2010) and single enrichment analysis implemented in the FatiGO module (Al‐Shahrour *et al*., 2004) for the proteomics data, both implemented in the Babelomics suite. KEGG annotation was extracted using REST services. For GSE logistic model performance, we used the following parameters: ‘Your annotation’ as database (KEGG pathways taken from REST service); adjusted p‐value by FDR procedure of Benjamini and Hochberg; and adjusted p‐value threshold of 0.05. For FatiGO, we used the following parameters: all genes annotated with KEGG as the reference set; ‘Your annotations’ as database (KEGG pathways taken from REST service); two‐tailed Fisher exact test; adjusted p‐value by FDR procedure of Benjamini and Hochberg; and adjusted p‐value threshold of 0.05.

The networks describing the lists of significantly abundant proteins in the proteomics experiment were calculated following the minimal connected network (MCN) methodology (Minguez *et al*., 2009). For the definition of the lists of proteins introduced in the network analysis, an extra threshold of fold change (FC)>1.2 was applied on the top of the p‐value cutoff. This FC was selected as having a good balance in both, setting a stricter statistical significance, and retrieving an optimal number of proteins for network analysis. The MCNs were built from custom scripts using STRING protein network (Szklarczyk *et al*., 2015) with a combined score ≥900.

### 2.8 Cell culture

The epithelial cell line NCM356 derived from the normal colon mucosa line was acquired under an MTA from InCell Corp. (San Antonio, TX, USA) and cultured at 37 °C in a humidified atmosphere of 95% air and 5% CO_2_ in Serum‐Free M3Base™ (Incell Corp.) media complemented with 10% fetal bovine serum (FBS) and a 1% penicillin/streptomycin. Cells were seeded into flasks and incubated with recommended culture medium and at diabetogenic glucose concentrations obtained by adding 24.5 mm d‐glucose to M3Base™, or osmotic control (obtained by adding 24.5 nM l‐Glucose to M3Base™), for five days. Then, cells were trypsinized at a 75–80% confluence and pellets were used for protein extraction and immunoblotting experiments.

### 2.9 Immunoblotting

Cytoplasmic and nuclear protein fractions were isolated using the NE‐PER kit (ThermoFisher), following the manufacturer's instructions. Proteins were quantified using a BCA kit (ThermoFisher), and 15 μg of protein from each fraction and condition was boiled with loading buffer at 95° C for 5 min and loaded into each well. Then, proteins underwent 10% polyacrylamide gel electrophoresis at 80 V and then at 120 V, followed by wet transfer onto nitrocellulose membranes at 100 V for 2 h. After blocking at room temperature with 5% skimmed milk for 1 h, primary antibodies against YAP (Clone D8H1X 1:1000; Cell Signaling Technologies, Danvers, MA, USA), TAZ (Clone V386 1 : 1000; Cell Signaling Technologies), and pan‐TEAD (Clone D3F7L 1 : 1000; Cell Signaling Technologies) were added, and membranes were incubated overnight at 4 °C. After rinsing with Tris‐buffered saline with Tween (TBST) three times for 5 min per wash, the corresponding secondary antibody was added and incubated for 1 h. Then, the membrane was washed three times for 5 min per wash and developed by chemiluminescence reagents. An Amersham Imager 600 chemiluminescence imager was used for high‐resolution digital imaging of proteins, and the gray values of the target bands were analyzed with imagej software (National Institutes of Health, Bethesda, MD, USA). These experiments were carried out in triplicate. β‐Actin (Sigma‐Aldrich) and lamin B1 (Abcam, Cambridge, UK) were used as cytoplasmic and nuclear loading controls, respectively. Differences among groups were studied by the Mann–Whitney *U*‐test.

## 3 Results

### 3.1 A highly homogeneous cohort of CC patients and a framework of multi‐omic data integration

**Figure 1:**
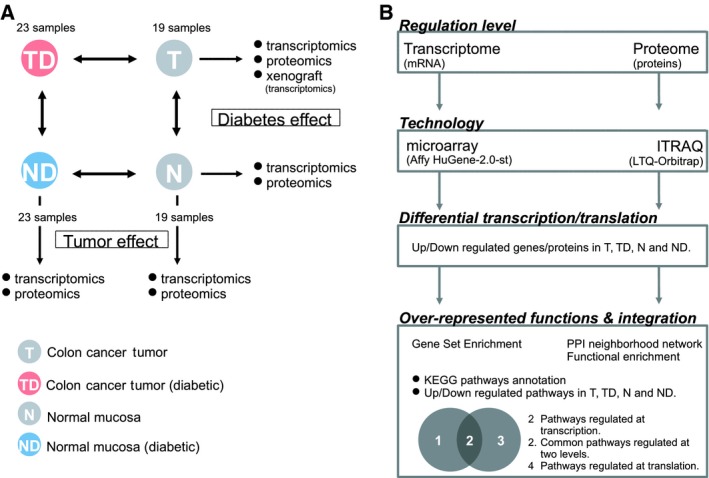
Experimental design and study road map. (A) Number of study samples (TD, tumor from diabetic patients; T, tumor from nondiabetic patients; ND, normal colonic mucosa from diabetic patients; N, normal colonic mucosa from nondiabetic patients) and studies performed. Comparisons performed between samples are noted by bidirectional arrows. Boxes indicate the biological aspect explored by each comparison. (B) Study road map.

A total of 42 patients were collected (19 nondiabetics and 23 with T2DM) with no difference in mutational status of usual CC biomarkers, nor in the proliferation index between the two classes (Table S1). Their clinicopathological features are depicted in Table 1. With this in hand, we aimed to explore the regulation of cancer‐related processes specific to T2DM. The global experimental design (Fig. 1A) included mRNA and protein expression analysis of four types of paired samples: (a) tumors from diabetic patients (TD), (b) normal colonic mucosa from diabetics (ND), (c) tumors from nondiabetics (T), and (d) normal colonic mucosa from nondiabetics (N). We identified upregulated genes/proteins and overrepresented processes in ND (compared to N) and in TD (compared to T). The high‐throughput transcriptomic and proteomic experiments performed to the same T, TD, N, and ND samples fed a systems biology inspired approach to finally extract the molecular pathways supported by the two regulation levels, mRNA and protein (Fig. 1B).

**Table 1:** Patient cohort description

Variable (*N*)	Selected patients (*N* = 42)	CC diabetic (*N* = 23)	CC nondiabetic (*N* = 19)
*Gender*			
Female	16	7	9
Male	26	16	10
*Grade*			
Low grade	9	4	5
High grade	33	19	14
*Tumor site*			
Right	21	13	8
Left	21	10	11
*Stage*			
Low	31	15	16
High	11	8	3
*Recurrence*			
Yes	6	4	2
No	36	19	17
*Final status*			
Alive	38	20	18
Dead	4	3	1
*Metformin*			
Yes	6	6	0
No	36	17	19

### 3.2 Tumor and normal colonic mucosa show a common signal in response to diabetes at the gene expression level

**Figure 2:**
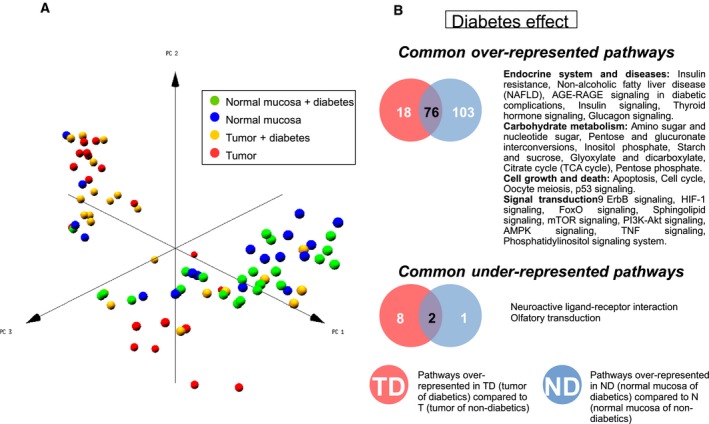
Sample distribution and pathways enriched in the transcriptomic analysis. (A) Principal component analysis of samples using microarray data. Samples are classified into normal colonic mucosa or tumor, from diabetic or nondiabetic patients. (B) Over‐ and underrepresented KEGG pathways in diabetic patients common to normal colonic mucosa and tumors.

Principal component analysis (PCA) of the normalized gene expression values of T, N, TD, and ND samples shows a clear separation between tumor and normal mucosa, but no observable differences between diabetics and nondiabetics either in tumors or in normal mucosa (Fig. 2A). These results are consistent with our previous clinical and epidemiological study using the same cohort (Prieto *et al*., 2017). Concurrently, a differential expression analysis of the transcriptomic data disclosed no up‐ or downregulated genes under T2DM conditions when false discovery rate (FDR)‐adjusted *P*‐value <0.05 was used. We performed gene set enrichment analysis searching for asymmetrical distributions of biological labels in an expression‐based ranked list of genes (Montaner and Dopazo, 2010), to the comparisons: (a) TD versus T and (b) ND versus N samples. Using the Kyoto Encyclopedia of Genes and Genomes (KEGG) as annotation, we extracted a total of 94 pathways overrepresented in TD compared to T, and 179 pathways overrepresented in ND compared to N (Fig. 2B). The overlap between both analyses (76 pathways) compiles altered processes under the diabetic condition that were common to the tumor and its adjacent mucosa. This level of overlap is significantly higher than expected by a random selection of same‐sized datasets of KEGG pathways (Fig. S1 and Materials and Methods) suggesting a similar behavior in both types of tissues under T2DM conditions.

Within these common pathways (Fig. 2B, Table S2), we found six related to the endocrine system including diabetes‐related processes. The metabolism of carbohydrates is also generally altered, with 7 routes overrepresented. Probably less expected is that diabetes seems to alter, in both the tumor and the normal mucosa, cell growth and death‐related processes and up to nine signal transduction pathways. As an external and independent comparison, we performed microarray experiments of human colon cancer xenografts in diabetic and nondiabetic mice (Prieto *et al*., 2017). In agreement with the human data, 50 out of the 76 common overrepresented pathways were also found overrepresented in the xenograft diabetic tumor compared to nondiabetic tumor, being mainly distributed in the same categories (Table S3).

Using a more specific and focused annotation based on curated pathways involved in inflammation (Loza *et al*., 2007), the two types of human diabetic samples (TD and ND) were found enriched in genes participating significantly in 4 processes (*Apoptosis*,* Glucocorticoid/PPAR*,* MAPK*, and *PI3K/AKT Signaling*) when compared to respective nondiabetic samples (T and N; Table S4). Here, the overlap between both comparisons was even larger, TD having only one other term enriched (*Adhesion‐Extravasation‐Migration*).

### 3.3 The core signaling of diabetes is different between tumor and mucosa at the protein level

**Figure 3:**
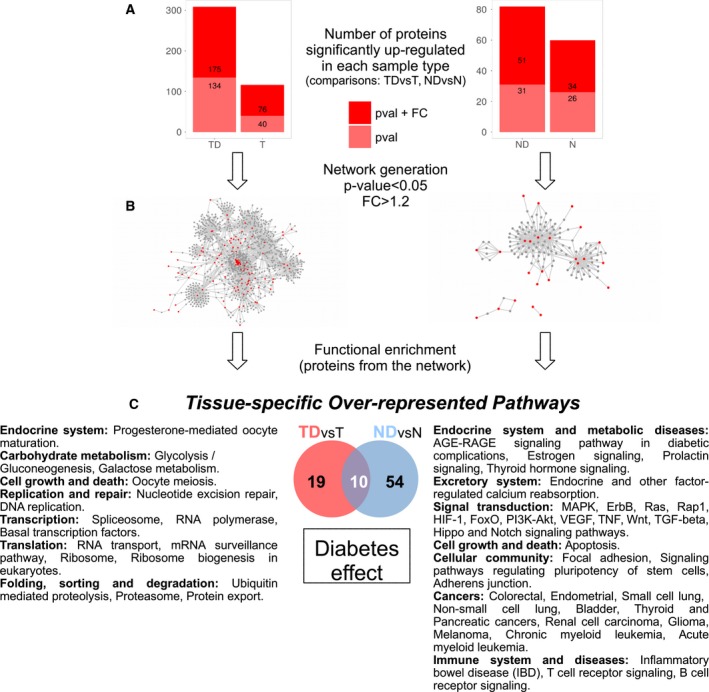
Differential analysis of protein abundance and overrepresented pathways in the proteomic analysis. (A) Protein abundance differential analysis in two comparisons: tumors from diabetics versus tumors from nondiabetics and normal colonic mucosa from diabetes versus normal colonic mucosa from nondiabetics. In light red, up‐ and downregulated proteins with a *P*‐value <0.05, and in dark, red proteins with *P*‐value <0.05 and FC > 1.2. (B) Networks generated from upregulated proteins (in red, FC > 1.2, *P*‐value <0.05) in diabetic patients’ tumor and normal colonic mucosa. (C) Differences in overrepresented KEGG pathways in tumors and normal colonic mucosa when comparing diabetic vs nondiabetic patients.

Using quantitative proteomic analysis, we performed two main comparisons to identify the effect of diabetes on tumor samples (TD versus T) and normal colonic mucosa (ND versus N). A total of 309 proteins were found significantly upregulated (116 downregulated) in TD versus T; 82 proteins were found upregulated (60 downregulated) in ND compared to N (Fig. 3A and Table S5). A classical functional enrichment (Al‐Shahrour *et al*., 2004) of KEGG pathways gave only one hit (*Ribosome*) overrepresented in TD compared to T and no hits in the rest of the possible comparisons. In a complementary approach, we defined the functional impact by first extracting the core of the signal and then zooming out using the interactome as propagation source. In this way, we mapped the upregulated proteins in TD and ND from the comparison with T and N samples, respectively (fold change (FC)>1.2), into the human interactome (Szklarczyk *et al*., 2015) and built the minimal network (Minguez *et al*., 2009) that interconnects them (Figs 3B and S2–S3). A functional enrichment analysis was then performed extracting the KEGG pathways that are influenced by the T2DM in tumor (Table S6) and its closer normal mucosa (Table S7).

A total of 29 and 64 pathways were overrepresented in TD and ND, respectively, 10 of them in common (Fig. 3C, Table S8). In contrast to the observations at the gene expression level, at the proteome level the overlap of pathways overrepresented was not so global (Fig. S1). This finding was already observed in the intersection between proteins upregulated in ND and TD (13 in common with FDR‐adjusted p‐value<0.05 and 6 selecting from them only those with FC>1.2). This might be due to several factors including the different approaches to extract the pathways or the fact that proteins are influenced by the environment more severely than gene expression.

Paying particular attention to pathways overrepresented specifically in diabetic tumors and mucosa (Fig. 3C), noncoincident processes related to hormone activity and others typically associated with hyperglycemia can be found in both types of samples (TD and ND). Surprisingly, the largest contrast is related to cancer or cancer‐associated processes. While in TD, the diabetic milieu affects mainly the cellular core machinery (replication, repair, transcription, translation, and protein folding and transport) that is already deregulated by the cancer itself, the mucosa (ND) network is enriched in several cancer‐linked specific pathways (13 signaling pathways and routes related to apoptosis and dedifferentiation; Fig. 3C).

### 3.4 Trends of carcinogenesis in the normal colonic mucosa under a diabetic environment

**Figure 4:**
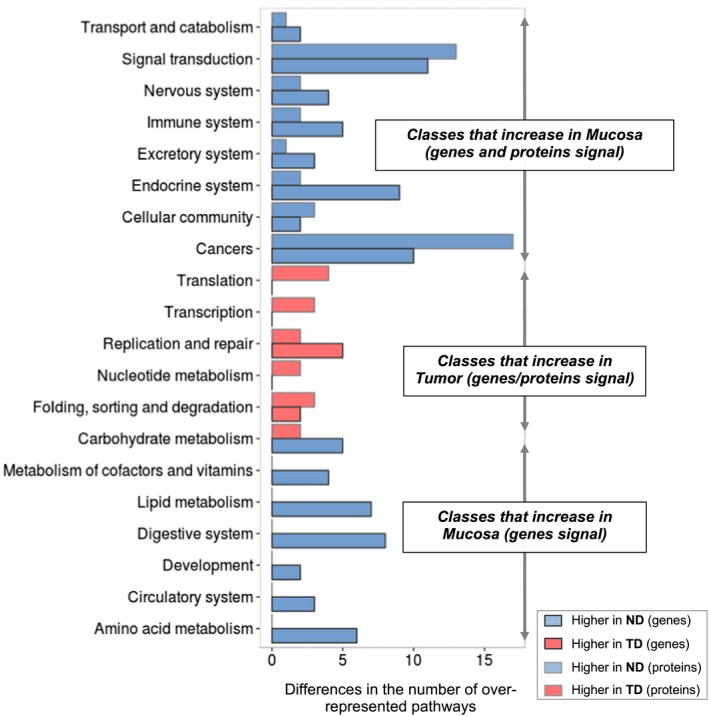
Global trends in processes overrepresented in diabetic samples at the transcriptional and translational levels. Differences in the number of KEGG pathways overrepresented in diabetic samples, classified in superclasses from two analyses (transcriptome and proteome). In light blue, the number of processes overrepresented in diabetic colonic mucosa (compared to nondiabetic colonic mucosa), and in light red, the number of processes overrepresented in diabetic tumor (compared to nondiabetic tumor). Dark borders represent the changes at translational level and light gray borders the changes at translational level.

In a broader analysis seeking for general trends and global deregulated processes, we grouped the KEGG pathways in superclasses and, per each, calculated the difference between the total number of pathways overrepresented in TD and ND, separating both regulatory levels, genes and proteins (Figs 4 and S4). Although this type of analysis cannot determine the grade of impact of T2DM (mild or serious), the difference of the total number of pathways overrepresented in ND and TD provides an overview on the global molecular fitness of the T2DM patients. Again, the effect of T2DM on tumors concentrates in core cellular processes (*Folding, sorting and degradation*,* Nucleotide metabolism*,* Replication and repair*,* Transcription*, and *Translation*), while in the normal mucosa, diabetes seems to fiddle with much more diverse and peripheral processes. Several of these globally affected superclasses are extracted only from the signal obtained by mRNAs (*Amino acid, Lipid* and *cofactors and vitamins metabolisms*,* Development*,* Circulatory*, and *Digestive systems*). Both regulatory levels (genes and proteins) agree in a wide effect of T2DM grouped in 8 global classes including the *Endocrine*,* Excretory*,* Immune* and *Nervous systems*,* Cellular community*, and *Transport and catabolism*. We remark here unforeseen big numbers of overrepresented pathways in ND compared to TD belonging to *Signal transduction and* cancer‐related processes where probably the latter are just a rearrangement of the former.

**Figure 5:**
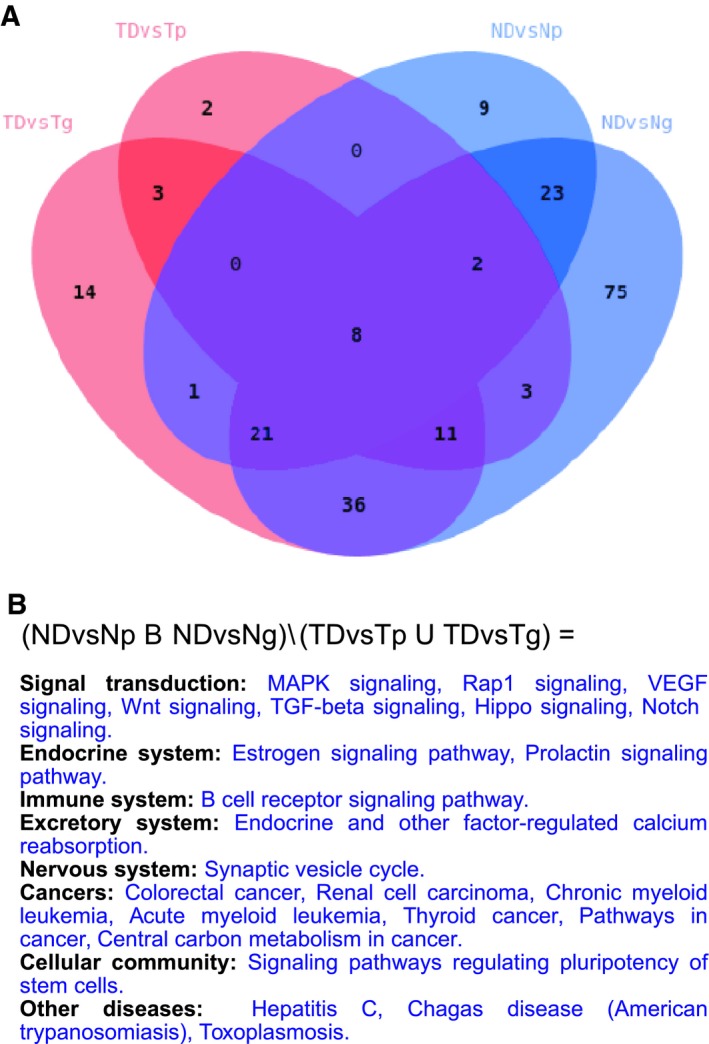
Integration of pathways overrepresented in diabetic samples in transcriptomic and proteomic analyses. (A) Overlap between KEGG pathways overrepresented in diabetics from all comparisons made in the study: (i) TDvsTg, tumor from diabetics versus tumor from nondiabetics at the transcriptome level; (ii) TDvsTp, tumor from diabetics versus tumor from nondiabetics at the proteome level; (iii) NDvsNp, normal colonic mucosa from diabetics versus normal colonic mucosa from nondiabetics at the proteome level; and (iv) NDvsNg, normal colonic mucosa from diabetics versus normal colonic mucosa from nondiabetics at the transcriptome level. (B) Details about the 23 pathways that are common to NDvsNp and NDvsNp comparisons and are not found in any of the TDvsT comparisons.

Our results point out indeed to a certain degree of deregulation of signaling and cancer‐related pathways in the normal mucosa in T2DM patients that is not present in nondiabetics. The common signal of diabetes in TD and ND has already been discussed; thus, we now focus on the pathways overrepresented exclusively in normal diabetic mucosa in both transcriptomic and proteomic experiments. From a total of 23 KEGG pathways fulfilling these requirements (Fig. 5A), and excluding processes that are rearrangements of core pathways (cancers, other diseases, and *Signaling pathways regulating pluripotency of stem cells* in the Cellular community superclass), 12 routes remain including 7 signaling pathways (*MAPK*,* Rap1*,* VEGF*,* Wnt*,* TGF‐*β, *Hippo*, and *Notch signaling*) and 3 related to the endocrine system (*Estrogen*,* Prolactin signaling pathways*, and *Endocrine and other factor‐regulated calcium reabsorption*); see Fig. 5B for a complete list.

### 3.5 Upregulation of the YAP/TAZ‐TEAD complex in glucose‐rich environment may trigger carcinogenesis

Transcriptome and proteome data identified 7 overrepresented signaling pathways in normal diabetic mucosa that have been previously associated with tumor development: MAPK, Rap1, VEGF, Wnt, TGF‐β, Hippo, and Notch signaling pathways. These pathways regulate common downstream effectors that control downstream transcriptomic programs that promote carcinogenesis (Hiemer *et al*., 2014; Hong *et al*., 2014; Konsavage *et al*., 2012; Slemmons *et al*., 2017; X. Wang *et al*., 2017).

**Figure 6:**
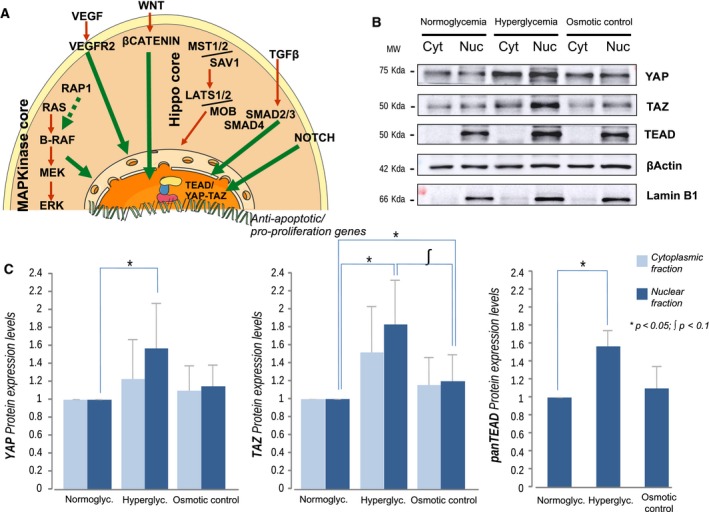
The TEAD/YAP‐TAZ axis is upregulated under hyperglycemia. (A) Molecular model where pathways found overrepresented in diabetic normal colonic mucosa but not in diabetic tumors converge in the TEAD/YAP‐TAZ axis. (B) Immunoblotting analysis of nontumor colon cultured cells. Three different culture conditions were assessed, representing normoglycemia, hyperglycemia, and osmotic control, for cytoplasm and nuclear samples. (C) Quantification of cell culture immunoblotting results. TEAD was only found in the nucleus. No differences were found among cytoplasmic fractions. Significance was calculated using the Mann–Whitney *U*‐test and 3 biologically independent replicates. Significance levels are represented by *(*P*‐value <0.05) and ʃ (*P*‐value <0.1). Error bars represent standard deviation.

To confirm a potential influence of hyperglycemia on normal colonic mucosa cells, we carried out *in vitro* experiments focused on the Hippo pathway, one to these overrepresented pathways which has been linked to carcinogenesis (Zanconato *et al*., 2016). The Hippo pathway leads to activation of YAP and TAZ that bind to the transcription factor TEAD. The YAP/TAZ‐TEAD core is closely connected to all seven overrepresented pathways and could be an important axis in triggering carcinogenesis in the diabetic colonic mucosa (Fig. 6A). To test the hypothesis that the key abnormality of diabetes, hyperglycemia, activates the YAP/TAZ‐TEAD in normal mucosa, we used an epithelial cell line (NCM356) derived from a normal colon mucosa line. We exposed the cell culture to a hyperglycemic environment and measured nuclear and cytoplasmic YAP, TAZ, and TEAD levels. Compared to normoglycemic conditions, exposure of cells to a high glucose concentration significantly increased the accumulation of YAP and TAZ proteins in the nuclear fraction. A similar effect was also observed in the transcriptional factor TEAD (Fig. 6B,C).

The osmotic control did not show significant differences compared to normoglycemia in nuclear YAP and TEAD levels, while an increase of TAZ was observed, still with lower levels than in hyperglycemia (Fig. 6B,C). No statistical differences were observed among cytoplasmic fractions. No differences in cell number or phenotype were observed in all tested groups (data not shown).

## 4 Discussion

We hypothesized that T2DM may provide a favorable molecular environment for CC tumorigenesis. An *in vitro* study has proposed T2DM to produce molecular changes in the colonic epithelium as a field cancerization driver (Rubin, 2013). Our work strengthens this idea with data obtained from human samples.

We designed an experimental and analysis framework to study the effect of T2DM in tumors and normal colonic mucosa. The cohort of patients was carefully designed in order to avoid a bias in tumor features, diabetes treatment, and BMI index between diabetics and nondiabetics (Prieto *et al*., 2017). The results were integrated and analyzed using a systems biology approach. The limitations of our work are various, including the particularities of formalin‐fixed paraffin‐embedded (FFPE) samples, reported to provide accurate but lower gene expression levels (Lüder Ripoli *et al*., 2016) and the expected low signal coming from the T2DM effect once the tumor is established (Prieto *et al*., 2017).

We identified cancer‐related routes targeted by the diabetic milieu in the normal mucosa which may have a key role in carcinogenesis. This signal is mostly shared with the impact of T2DM within the tumor. Thus, among overexpressed pathways common to both tumor and mucosa, we found the mammalian target of rapamycin (mTOR), AMP‐activated kinase (AMPK), and tumor necrosis factor (TNF) signaling, already proposed to link diabetes, obesity, and cancer (Jurjus *et al*., 2016; Yao *et al*., 2014). Other pathways may also play a role in both diseases. Thus, protein kinase B (AKT) phosphorylation by mTOR mediated by phosphatidylinositol is central to insulin regulation (Mackenzie and Elliott, 2014). Upstream to mTOR activation, the Forkhead box protein (FoxOs) family is associated with glucose intolerance (Tsuchiya and Ogawa, 2017) as well as with CC tumorigenesis promotion (Cui *et al*., 2014). Also hypoxia may play a role in tumorigenesis in CC (Tan *et al*., 2017) and was reported to be a risk factor for diabetic retinopathy in combination with hyperglycemia (Arden and Sivaprasad, 2011). In the same way, sphingolipids are known to mediate insulin resistance (Russo *et al*., 2013) and interact with the Wnt/β‐catenin pathway in CC (García‐Barros *et al*., 2014). Other processes altered under diabetic conditions point to a regulation of carcinogenesis, as the epidermal growth factor receptor (ErbB) and the PI3K–Akt signaling pathways, both in the upstream cascade of mTOR, and regulators of proliferation in several cancers including CC (Herbst, 2004). An overrepresentation of apoptosis regulated pathways had been previously observed in a human transcriptomic analysis of another target organ of diabetes, the kidney (Sanchez‐Niño *et al*., 2010).

The diabetic normal mucosa shows deregulated processes that have been functionally related to initiators of tumorigenesis. The seven signaling processes overrepresented uniquely in normal diabetic mucosa in both proteomic and transcriptomic studies merit special attention as they can point to a field cancerization driven by T2DM. Some of them have already been linked to diabetes and cancer. Protein Epac2 activates the small GTPase Rap1 in pancreatic beta cells and is implicated in the regulation of insulin (Shibasaki *et al*., 2007) and cell invasion and metastasis (Zhang *et al*., 2017). Activation of these small GTPases ultimately leads to ERK1/2, JNK1, and p38 phosphorylation, members of the MAP kinases. This increased phosphorylation has been reported in diabetics compared to healthy subjects (Fröjdö *et al*., 2009) and is largely involved in cell proliferation (Mebratu and Tesfaigzi, 2009; Wagner and Nebreda, 2009). Upstream effectors of this signaling cascade include deregulation of the TGF‐β pathway, increased in T2DM patients (Qiao *et al*., 2017) and described as a CC oncogene (Xu and Pasche, 2007); and Wnt activation, postulated as a possible link between diabetes and cancer (García‐Jiménez *et al*., 2014). Two additional pathways cooperate with Wnt in CC tumorigenesis: Hippo (Rosenbluh *et al*., 2012), proposed as potential target in diabetes (S.‐P. Wang and Wang, 2016), and Notch (Vinson *et al*., 2016) that plays an important role in B cells under hyperglycemia (Darville and Eizirik, 2006). Lastly, angiogenesis resulting from VEGF activation is known to contribute to cancer and to diabetes complications and recent evidences indicate an angiogenic switch mediated by VEGF in premalignant tissues such as atypical colon adenomas (Cheng and Ma, 2015).

In view of our results, we selected specific targets to confirm hyperglycemia influence. Many evidences suggested a role in cancer initiation of the protein complex YAP/TAZ‐TEAD (Zanconato *et al*., 2016) including several signaling pathways that we found altered in normal diabetic mucosa: MAPK (Hong *et al*., 2014), VEGF (X. Wang *et al*., 2017), Wnt (Konsavage *et al*., 2012), TGF‐β (Hiemer *et al*., 2014), or the Notch pathway (Slemmons *et al*., 2017). We observed activation of the YAP/TAZ‐TEAD axis under hyperglycemic conditions in the assay of cultured normal colon epithelial cells. These promising preliminary results could be the starting point for further efforts to clarify the role of YAP/TAZ‐TEAD, and other molecular effectors, in the molecular scenario responsible for the field cancerization associated with T2DM in CC patients.

## 5 Conclusions

We describe here for the first time a number of cancer‐related processes deregulated by diabetes in normal colon mucosa adjacent to tissue which has undergone malignant transformation. Those molecular changes taking place already in normal mucosa bring to light what can be an influence area, responsible for the reported increased risk for CC development of diabetic patients. These results are nested within the concept of field of cancerization, a well‐established paradigm that we propose to be modulated by T2DM. The clinical impact derived from these data could involve the T2DM patient management and also open a new framework in the study of the cancer risk associated with diabetes.

## 7 Conflicts of Interest

The authors have no conflicts of interest to declare.

## 8 Author contributions

LDP, PM, and GAL wrote the manuscript and researched data. LDP, PM, GAL, FR, JE, and JGF conceived and designed the experiments. MC, SSC, IP, PGA, CV, SPN, and NG researched data. SM, ABS, OA, CGG, PE, FV, CA, AO, FR, JE, and JGF contributed to discussion and reviewed/edited the manuscript.

## Supporting information


**Table S1.** Mutational, immunohistochemical, and proliferation characterization of samples.
**Table S2.** Common overrepresented pathways in diabetic conditions in two comparisons: (i) tumors from diabetic patients versus tumors from nondiabetic patients and (ii) normal colonic mucosas from diabetic patients versus normal colonic mucosas from nondiabetic patients.
**Table S3.** Common overrepresented pathways in diabetic conditions in tumors and normal mucosas from human and xenograft samples.
**Table S4.** Overrepresented pathways involved in inflammation (24) in diabetic conditions in two comparisons: (i) tumors from diabetic patients versus tumors from nondiabetic patients and (ii) normal colonic mucosas from diabetic patients versus normal colonic mucosas from nondiabetic patients. Pathways in common between the comparisons are colored in light gray.
**Table S5.** Proteins up‐ and downregulated in two tumors and normal colonic mucosas from patients with diabetes. TD means tumors from diabetic patients. T, tumors from nondiabetic patients. ND, normal colonic mucosa from diabetic patients. And N, normal colonic mucosa from nondiabetic patients.
**Table S6.** Overrepresented KEGG pathways in the network made with differentially upregulated proteins in tumors from diabetic patients compared to tumors from nondiabetic patients.
**Table S7.** Overrepresented KEGG pathways in the network made with differentially upregulated proteins in normal colonic mucosa from diabetic patients compared to normal colonic mucosa from nondiabetic patients.
**Table S8.** Overrepresented KEGG pathways in tumors from diabetic patients (TD) and normal colonic mucosas from diabetic patients (ND) compared to tumors from nondiabetic patients (T) and normal colonic mucosas from nondiabetic patients (N), respectively.
**Fig. S1.** Overlap of KEGG pathways overrepresented comparing (i) tumors from diabetic patients to tumors from nondiabetic patients; and (ii) adjacent mucosa from diabetic patients to adjacent mucosa from nondiabetic patients.
**Fig. S2.** Minimal connected network of proteins upregulated in tumors from diabetic patients (TD) compared to tumors from nondiabetic patients (T). In red, differentially expressed proteins with a fold change > 1.2 and a p‐value <0.05 in diabetic vs nondiabetic conditions.
**Fig. S3.** Minimal connected network of proteins upregulated in adjacent mucosa from diabetic patients (ND) compared to adjacent mucosa from nondiabetic patients (N). In red, differentially expressed proteins with a fold change > 1.2 and a *P*‐value <0.05 in diabetic vs nondiabetic conditions.
**Fig. S4.** (A,B,C) General trends in deregulation of genes and proteins in diabetic tumor and diabetic mucosa.
