# Supplementary material for: Molecular evidence of field cancerization initiated by diabetes in colon cancer patients
Source: Mol Oncol. 2019 Feb 16;13(4):857–72. doi: 10.1002/1878-0261.12438 (PMC6441931; doi:10.1002/1878-0261.12438)
Supplement: Supplementary file 1 — Table S1. Mutational, immunohistochemical, and proliferation characterization of samples. Table S2. Common overrepresented pathways in diabetic conditions in two comparisons: (i) tumors from diabetic patients versus tumors from nondiabetic patients and (ii) normal colonic mucosas from diabetic patients versus normal colonic mucosas from nondiabetic patients. Table S3. Common overrepresented pathways in diabetic conditions in tumors and normal mucosas from human and xenograft samples. Table S4. Overrepresented pathways involved in inflammation (24) in diabetic conditions in two comparisons: (i) tumors from diabetic patients versus tumors from nondiabetic patients and (ii) normal colonic mucosas from diabetic patients versus normal colonic mucosas from nondiabetic patients. Pathways in common between the comparisons are colored in light gray. Table S5. Proteins up‐ and downregulated in two tumors and normal colonic mucosas from patients with diabetes. TD means tumors from diabetic patients. T, tumors from nondiabetic patients. ND, normal colonic mucosa from diabetic patients. And N, normal colonic mucosa from nondiabetic patients. Table S6. Overrepresented KEGG pathways in the network made with differentially upregulated proteins in tumors from diabetic patients compared to tumors from nondiabetic patients. Table S7. Overrepresented KEGG pathways in the network made with differentially upregulated proteins in normal colonic mucosa from diabetic patients compared to normal colonic mucosa from nondiabetic patients. Table S8. Overrepresented KEGG pathways in tumors from diabetic patients (TD) and normal colonic mucosas from diabetic patients (ND) compared to tumors from nondiabetic patients (T) and normal colonic mucosas from nondiabetic patients (N), respectively. Fig. S1. Overlap of KEGG pathways overrepresented comparing (i) tumors from diabetic patients to tumors from nondiabetic patients; and (ii) adjacent mucosa from diabetic patients to adjacent mucosa from nondia [file MOL2-13-857-s001.pdf]

Supporting Information for

Molecular evidences of field cancerization initiated by diabetes in colon cancer patients

Laura Del Puerto-Nevado 1*, Pablo Minguez 2*#, Marta Corton 2,3, Sonia Solanes-Casado 1, Isabel Prieto 4, Sebastian Mas 5, Ana Belen Sanz 6,7, Paula Gonzalez-Alonso 8, Cristina Villaverde 2,3, Sergio Portal-Nuñez 9,10, Oscar Aguilera 1, Carmen Gomez-Guerrero 5, Fernando Vivanco 11, Pedro Esbrit 9, Nieves Gonzalez 5, Carmen Ayuso 2,3, Alberto Ortiz 6,7, Federico Rojo 8, Jesús Egido 5, Gloria Alvarez-Llamas 7,11#, Jesus Garcia-Foncillas 1 on behalf of the DiabetesCancerConnect Consortium.

1 Translational Oncology Division, Oncohealth Institute, IIS-Fundacion Jimenez Diaz-UAM, Madrid, Spain. 2 Genetics Departement, IIS-Fundacion Jimenez Diaz-UAM, Madrid, Spain. 3 Center for Biomedical Network Research on Rare Diseases (CIBERER), ISCIII, Madrid, Spain. 4 Radiation Oncology, Oncohealth Institute, IIS-Fundacion Jimenez Diaz-UAM, Madrid, Spain. 5 Renal, Vascular and Diabetes Research Laboratory, IIS-Fundacion Jimenez Diaz-UAM, Spanish Biomedical Research Network in Diabetes and Associated Metabolic Disorders (CIBERDEM), Madrid, Spain. 6 Nephrology and Hypertension Department, IIS-Fundacion Jimenez Diaz-UAM, Madrid, Spain. 7 REDINREN, Madrid, Spain. 8 Pathology Department, IIS-Fundacion Jimenez Diaz-UAM, Madrid, Spain. 9 Bone and Mineral Metabolism Laboratory, IIS-Fundacion Jimenez Diaz-UAM, Madrid, Spain. 10 Applied Molecular Medicine Institute, School of Medicine, Universidad San Pablo CEU, CEU Universities, Madrid, Spain. 11 Immunology Department, IIS-Fundacion Jimenez Diaz-UAM, Madrid, Spain.

*These authors contributed equally to this work; #Corresponding authors: pablo.minguez@quironsalud.es; galvarez@fjd.es

This PDF file includes:
Tables S1 to S8
Figures S1 to S4

Table S1. Mutational, immunohistochemical and proliferation characterization of samples.

	Non-diabetics		Diabetics		
Gene sequencing	WT	Mutant	WT	Mutant	p-value *(Fisher's Exact test)*
KRAS	14	9	14	9	1
NRAS	21	2	23	0	0.49
BRAF	21	2	20	3	1
Immunohistochemical analysis of MMR genes	Negative	Positive	Negative	Positive	
MLH1	6	17	3	20	0.46
MSH2	1	22	0	23	1
MSH6	1	22	0	23	1
PSM2	6	17	3	20	0.46
Proliferation analysis	Low	High	Low	High	
Ki-67	4	15	8	11	0.29

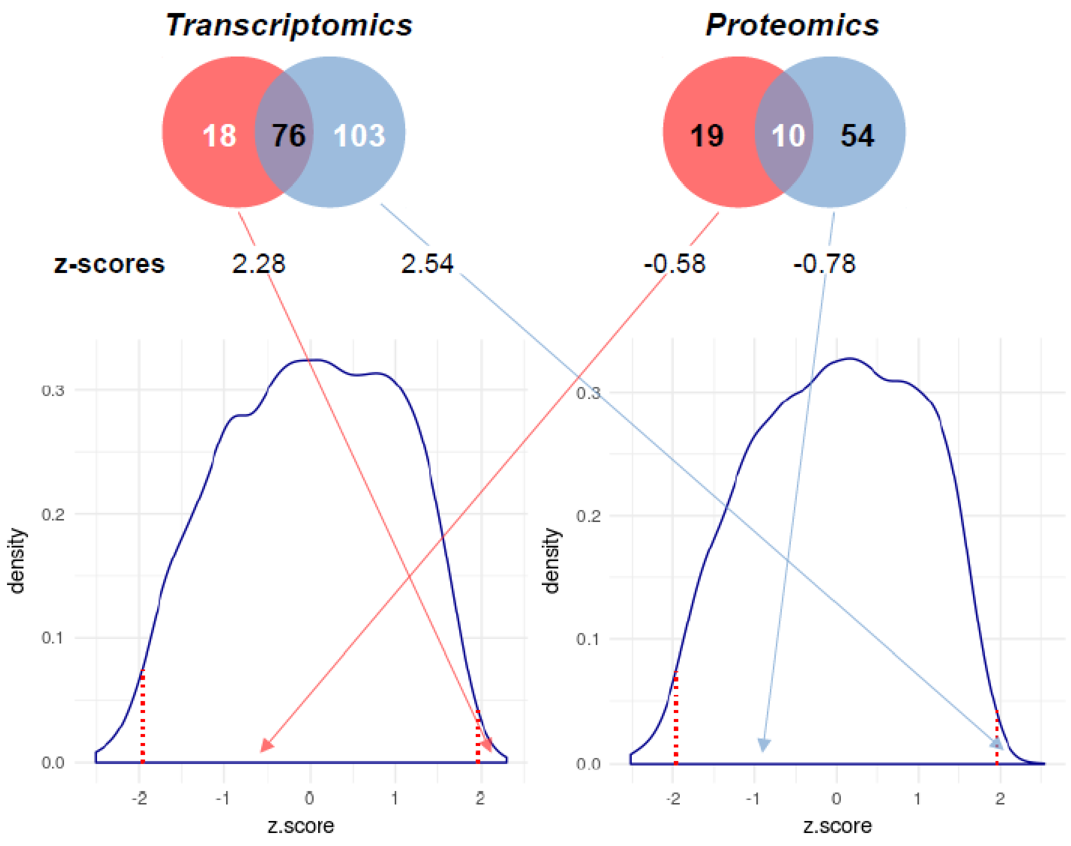

Figure S1. Overlap of KEGG pathways over-represented comparing: i) tumors from diabetic patients to tumors from non-diabetic patients; and ii) adjacent mucosa from diabetic patients to adjacent mucosa from non-diabetic patients.

Table S2. Common over-represented pathways in diabetic conditions in two comparisons: i) tumors from diabetic patients versus tumors from non-diabetic patients and ii) normal colonic mucosas from diabetic patients versus normal colonic mucosas from non-diabetic patients.

KEGG id	KEGG pathway name	Super-class
hsa01100	Metabolic pathways	Global and overview maps
hsa00230	Purine metabolism	Nucleotide metabolism
hsa04012	ErbB signaling pathway	Signal transduction
hsa04066	HIF-1 signaling pathway	Signal transduction
hsa04068	FoxO signaling pathway	Signal transduction
hsa04071	Sphingolipid signaling pathway	Signal transduction
hsa04150	mTOR signaling pathway	Signal transduction
hsa04151	PI3K-Akt signaling pathway	Signal transduction
hsa04152	AMPK signaling pathway	Signal transduction
hsa04210	Apoptosis	Cell growth and death
hsa04211	Longevity regulating pathway - mammal	Aging

hsa04510	Focal adhesion	Cellular community
hsa04530	Tight junction	Cellular community
hsa04660	T cell receptor signaling pathway	Immune system
hsa04666	Fc gamma R-mediated phagocytosis	Immune system
hsa04668	TNF signaling pathway	Signal transduction
hsa04722	Neurotrophin signaling pathway	Nervous system
hsa04910	Insulin signaling pathway	Endocrine system
hsa04919	Thyroid hormone signaling pathway	Endocrine system
hsa04922	Glucagon signaling pathway	Endocrine system
hsa04931	Insulin resistance	Endocrine and metabolic diseases
hsa04932	Non-alcoholic fatty liver disease (NAFLD)	Endocrine and metabolic diseases
hsa04933	AGE-RAGE signaling pathway in diabetic complications	Endocrine and metabolic diseases
hsa05152	Tuberculosis	Infectious diseases: Bacterial
hsa05161	Hepatitis B	Infectious diseases: Viral
hsa05164	Influenza A	Infectious diseases: Viral
hsa05166	HTLV-I infection	Infectious diseases: Viral
hsa05169	Epstein-Barr virus infection	Infectious diseases: Viral
hsa05205	Proteoglycans in cancer	Cancers: Overview
hsa05213	Endometrial cancer	Cancers: Specific types
hsa05222	Small cell lung cancer	Cancers: Specific types
hsa00520	Amino sugar and nucleotide sugar metabolism	Carbohydrate metabolism
hsa03013	RNA transport	Translation
hsa05203	Viral carcinogenesis	Cancers: Overview
hsa04144	Endocytosis	Transport and catabolism
hsa05130	Pathogenic Escherichia coli infection	Infectious diseases: Bacterial
hsa04141	Protein processing in endoplasmic reticulum	Folding, sorting and degradation
hsa05010	Alzheimer's disease	Neurodegenerative diseases
hsa00240	Pyrimidine metabolism	Nucleotide metabolism
hsa03015	mRNA surveillance pathway	Translation
hsa03010	Ribosome	Translation
hsa04142	Lysosome	Transport and catabolism
hsa00190	Oxidative phosphorylation	Energy metabolism
hsa05012	Parkinson's disease	Neurodegenerative diseases
hsa05016	Huntington's disease	Neurodegenerative diseases
hsa03040	Spliceosome	Transcription
hsa04120	Ubiquitin mediated proteolysis	Folding, sorting and degradation
hsa00970	Aminoacyl-tRNA biosynthesis	Translation
hsa05100	Bacterial invasion of epithelial cells	Infectious diseases: Bacterial
hsa05131	Shigellosis	Infectious diseases: Bacterial
hsa04520	Adherens junction	Cellular community
hsa04110	Cell cycle	Cell growth and death
hsa04114	Oocyte meiosis	Cell growth and death
hsa04115	p53 signaling pathway	Cell growth and death

hsa05168	Herpes simplex infection	Infectious diseases: Viral
hsa03008	Ribosome biogenesis in eukaryotes	Translation
hsa00510	N-Glycan biosynthesis	Glycan biosynthesis and metabolism
hsa05120	Epithelial cell signaling in Helicobacter pylori infection	Infectious diseases: Bacterial
hsa03022	Basal transcription factors	Transcription
hsa00270	Cysteine and methionine metabolism	Amino acid metabolism
hsa01230	Biosynthesis of amino acids	Global and overview maps
hsa04145	Phagosome	Transport and catabolism
hsa05110	Vibrio cholerae infection	Infectious diseases: Bacterial
hsa00040	Pentose and glucuronate interconversions	Carbohydrate metabolism
hsa04621	NOD-like receptor signaling pathway	Immune system
hsa04070	Phosphatidylinositol signaling system	Signal transduction
hsa00562	Inositol phosphate metabolism	Carbohydrate metabolism
hsa04962	Vasopressin-regulated water reabsorption	Excretory system
hsa00500	Starch and sucrose metabolism	Carbohydrate metabolism
hsa01200	Carbon metabolism	Global and overview maps
hsa03060	Protein export	Folding, sorting and degradation
hsa00630	Glyoxylate and dicarboxylate metabolism	Carbohydrate metabolism
hsa00480	Glutathione metabolism	Metabolism of other amino acids
hsa00020	Citrate cycle (TCA cycle)	Carbohydrate metabolism
hsa01210	2-Oxocarboxylic acid metabolism	Global and overview maps
hsa00030	Pentose phosphate pathway	Carbohydrate metabolism

Table S3. Common over-represented pathways in diabetic conditions in tumors and normal mucosas from human and xenograft samples. The table is composed by the pathways that have fulfilled three conditions: 1) over-represented in tumors from diabetic patients versus tumors from non-diabetic patients; 2) over-represented normal colonic mucosas from diabetic patients versus normal colonic mucosas from non-diabetic patients; 3) over-represented in tumors from diabetic xenografts versus tumors from non-diabetic xenografts.

KEGG id	KEGG pathway name	Super-class
hsa01100	Metabolic pathways	Global and overview maps
hsa00230	Purine metabolism	Nucleotide metabolism
hsa04012	ErbB signaling pathway	Signal transduction
hsa04152	AMPK signaling pathway	Signal transduction
hsa04211	Longevity regulating pathway - mammal	Aging
hsa04722	Neurotrophin signaling pathway	Nervous system
hsa04910	Insulin signaling pathway	Endocrine system
hsa04922	Glucagon signaling pathway	Endocrine system
hsa04931	Insulin resistance	Endocrine and metabolic diseases
hsa04932	Non-alcoholic fatty liver disease (NAFLD)	Endocrine and metabolic diseases
hsa05169	Epstein-Barr virus infection	Infectious diseases: Viral
hsa00520	Amino sugar and nucleotide sugar metabolism	Carbohydrate metabolism

hsa03013	RNA transport	Translation
hsa05203	Viral carcinogenesis	Cancers: Overview
hsa04144	Endocytosis	Transport and catabolism
hsa04141	Protein processing in endoplasmic reticulum	Folding, sorting and degradation
hsa05010	Alzheimer's disease	Neurodegenerative diseases
hsa00240	Pyrimidine metabolism	Nucleotide metabolism
hsa03015	mRNA surveillance pathway	Translation
hsa03010	Ribosome	Translation
hsa04142	Lysosome	Transport and catabolism
hsa00190	Oxidative phosphorylation	Energy metabolism
hsa05012	Parkinson's disease	Neurodegenerative diseases
hsa05016	Huntington's disease	Neurodegenerative diseases
hsa03040	Spliceosome	Transcription
hsa04120	Ubiquitin mediated proteolysis	Folding, sorting and degradation
hsa00970	Aminoacyl-tRNA biosynthesis	Translation
hsa05131	Shigellosis	Infectious diseases: Bacterial
hsa04110	Cell cycle	Cell growth and death
hsa04114	Oocyte meiosis	Cell growth and death
hsa04115	p53 signaling pathway	Cell growth and death
hsa05168	Herpes simplex infection	Infectious diseases: Viral
hsa03008	Ribosome biogenesis in eukaryotes	Translation
hsa00510	N-Glycan biosynthesis	Glycan biosynthesis and metabolism
hsa05120	Epithelial cell signaling in Helicobacter pylori infection	Infectious diseases: Bacterial
hsa03022	Basal transcription factors	Transcription
hsa00270	Cysteine and methionine metabolism	Amino acid metabolism
hsa01230	Biosynthesis of amino acids	Global and overview maps
hsa00040	Pentose and glucuronate interconversions	Carbohydrate metabolism
hsa04070	Phosphatidylinositol signaling system	Signal transduction
hsa00562	Inositol phosphate metabolism	Carbohydrate metabolism
hsa04962	Vasopressin-regulated water reabsorption	Excretory system
hsa00500	Starch and sucrose metabolism	Carbohydrate metabolism
hsa01200	Carbon metabolism	Global and overview maps
hsa03060	Protein export	Folding, sorting and degradation
hsa00630	Glyoxylate and dicarboxylate metabolism	Carbohydrate metabolism
hsa00480	Glutathione metabolism	Metabolism of other amino acids
hsa00020	Citrate cycle (TCA cycle)	Carbohydrate metabolism
hsa01210	2-Oxocarboxylic acid metabolism	Global and overview maps
hsa00030	Pentose phosphate pathway	Carbohydrate metabolism

Table S4. Over-represented pathways involved in inflammation (24) in diabetic conditions in two comparisons: i) tumors from diabetic patients versus tumors from non-diabetic patients and ii) normal colonic mucosas from diabetic patients versus normal colonic mucosas from non-diabetic patients. Pathways in common between the comparisons are colored in light gray.

Over-represented inflammation pathways in tumors from diabetic patients versus tumors from non-diabetic patients

Pathway name (involved in inflammation)	Annotated genes	FDR adjusted p-value
Adhesion-Extravasation-Migration	PTPRU,VCAM1,CD58,CD2,MUC1,CD48,F11R,XCL1,SELP,SELL,SELE,RASSF5,CD34,ITGB1,CXCL12,VCL,ADAM8,ARHGAP1,SIPA1,CTTN,FUT4,MMP7,MMP10,MMP1,MMP12,THY1,JAM3,CD9,ITGB7,ITGA5,MMP19,MYL6,SELPLG,PXN,MMP14,SPN,ITGAL,ITGAM,ITGAX,ITGAD,MMP2,CCL22,CX3CL1,CCL17,CDH5,CRK,ITGAE,CXCL16,MYH10,CCL2,CCL11,CCL8,CCL13,CCL1,CCL5,CCL16,CCL23,CCL4,CCR7,ITGA2B,ITGB3,ITGA3,ICAM2,PECAM1,ITGB4,CD226,ICAM1,ICAM5,ICAM3,CEACAM5,CEACAM6,CEACAM1,CEACAM8,PLAUR,VASP,CD33,ROCK2,ITGA6,ITGA4,ITGAV,ALS2,CCL20,SIGLEC1,MMP9,JAM2,ITGB2,MYH9,CCR4,CX3CR1,CCR8,CXCR6,XCR1,CCR1,CCR3,CCR2,CCR5,CCRL2,RHOA,ALCAM,CD47,CD96,RHOH,PPBP,CXCL5,CXCL3,CXCL2,CXCL9,CXCL10,CXCL11,CXCL13,FYB,CCL28,ITGA2,CXCL14,LECT2,HMMR,NT5E,CCR6,CCL26,CCL24,CD36,CCL19,CCL21,CD99	0.04
Apoptosis Signaling	DFFB,DFFA,CASP9,MCL1,DAP3,LMNA,FASLG,CAPN2,PARP1,FAS,CASP7,GAS2,BAD,CAPN1,FADD,BIRC3,BIRC2,CASP12,BCL2L14,CRADD,APAF1,DIABLO,ACIN1,RIPK3,BCL2L10,BCL2A1,TP53,ROCK1,BCL2,CASP14,CAPNS1,BAX,BCL2L12,HTRA2,BCL2L11,TANK,CFLAR,CASP10,CASP8,CAPN10,BCL2L1,BCL2L13,A4GALT,BIK,FAIM,PTPN13,CASP6,CASP3,DAP,DAXX,BAK1,TNFRSF21,CASP8AP2,CYCS,CASP2,TNFRSF10B,TNFRSF10D,TNFRSF10A,DAPK1,SPTAN1,ENDOG	0.003
Glucocorticoid/PPAR signaling	GMEB1,FAF1,FKBP4,NR4A1,NCOA1,NR4A2,SDPR,HSPD1,GMEB2,NRIP1,PPARA,KPNA1,NR3C1,CITED2,GLCCI1,NCOA2	0.039
MAPK signaling	PRKCZ,RAP1GAP,RPS6KA1,MAP3K6,HDAC1,MKNK1,JUN,RAP1A,NRAS,SHC1,MEF2D,IFI16,PLA2G4A,MAPKAPK2,ATF3,DUSP10,PRKCQ,MAPK8,HRAS,MADD,RPS6KA4,FOSL1,PPP1CA,PAK1,PPP2R1B,ETS1,KRAS,RAPGEF3,ATF1,DDIT3,DUSP6,PPP1CC,MAPKAPK5,SOS2,PPM1A,MAX,FOS,RPS6KA5,RASGRP1,TLN2,MAP2K1,MEF2A,MAPK3,BCAR1,MAP2K4,KSR1,PRKCA,MAP2K6,MAP2K2,JUND,MAP4K1,PPP2R1A,YWHAQ,MYCN,PPP1CB,SOS1,PRKCE,DUSP2,ATF2,PPP1R7,SRC,PLCG1,YWHAB,ETS2,HMGN1,MAPK1,YWHAH,ATF4,EP300,MAPK12,MAPK11,PPARG,RAF1,KCNH8,MAPKAPK3,PRKCD,PPP2R3A,EGF,MAP3K1,RASA1,MEF2C,HINT1,PPP2CA,PPP2R2B,DUSP1,MAPK9,MAPK14,MAPK13,MAP3K7,HDAC2,PTPRK,MAP3K5,ESR1,RAC1,EGFR,HSPB1,YWHAG,DUSP4,PPP2CB,LYN,YWHAZ,MYC,PTK2,TLN1,RAPGEF1,ARAF,ELK1,DUSP9	2.820E-5
PI3K/AKT Signaling	PIK3CD,PIK3R3,THEM4,AKT3,MAP3K8,ILK,RPS6KB2,CCND1,INPPL1,CDKN1B,MDM2,HSP90AA1,AKT1,TSC2,PDPK1,PIK3R5,MYH4,RPS6KB1,CDC37,PIK3R2,AKT2,INPP5D,CTNNB1,PIK3CB,PIK3CA,EIF4E,GAB1,PIK3R1,CDKN1A,NOS3,RHEB,TSC1	0.004

Over-represented inflammation pathways in normal colonic mucosa from diabetic patients versus normal colonic mucosa from non-diabetic patients

Pathway name (involved in inflammation)	Annotated genes	FDR adjusted p-value
Apoptosis Signaling	DFFB,DFFA,CASP9,MCL1,DAP3,LMNA,FASLG,CAPN2,PARP1,FAS,CASP7,GAS2,BAD,CAPN1,FADD,BIRC3,BIRC2,CASP12,BCL2L14,CRADD,APAF1,DIABLO,ACIN1,RIPK3,BCL2L10,BCL2A1,TP53,ROCK1,BCL2,CASP14,CAPNS1,BAX,BCL2L12,HTRA2,BCL2L11,TANK,CFLAR,CASP10,CASP8,CAPN10,BCL2L1,BCL2L13,A4GALT,BIK,FAIM,PTPN13,CASP6,CASP3,DAP,DAXX,BAK1,TNFRSF21,CASP8AP2,CYCS,CASP2,TNFRSF10B,TNFRSF10D,TNFRSF10A,DAPK1,SPTAN1,ENDOG	0.002
Glucocorticoid/PPAR signaling	GMEB1,FAF1,FKBP4,NR4A1,NCOA1,NR4A2,SDPR,HSPD1,GMEB2,NRIP1,PPARA,KPNA1,NR3C1,CITED2,GLCCI1,NCOA2	0.001
MAPK signaling	PRKCZ,RAP1GAP,RPS6KA1,MAP3K6,HDAC1,MKNK1,JUN,RAP1A,NRAS,SHC1,MEF2D,IFI16,PLA2G4A,MAPKAPK2,ATF3,DUSP10,PRKCQ,MAPK8,HRAS,MADD,RPS6KA4,FOSL1,PPP1CA,PAK1,PPP2R1B,ETS1,KRAS,RAPGEF3,ATF1,DDIT3,DUSP6,PPP1CC,MAPKAPK5,SOS2,PPM1A,MAX,FOS,RPS6KA5,RASGRP1,TLN2,MAP2K1,MEF2A,MAPK3,BCAR1,MAP2K4,KSR1,PRKCA,MAP2K6,MAP2K2,JUND,MAP4K1,PPP2R1A,YWHAQ,MYCN,PPP1CB,SOS1,PRKCE,DUSP2,ATF2,PPP1R7,SRC,PLCG1,YWHAB,ETS2,HMGN1,MAPK1,YWHAH,ATF4,EP300,MAPK12,MAPK11,PPARG,RAF1,KCNH8,MAPKAPK3,PRKCD,PPP2R3A,EGF,MAP3K1,RASA1,MEF2C,HINT1,PPP2CA,PPP2R2B,DUSP1,MAPK9,MAPK14,MAPK13,MAP3K7,HDAC2,PTPRK,MAP3K5,ESR1,RAC1,EGFR,HSPB1,YWHAG,DUSP4,PPP2CB,LYN,YWHAZ,MYC,PTK2,TLN1,RAPGEF1,ARAF,ELK1,DUSP9	2.946E-7
PI3K/AKT Signaling	PIK3CD,PIK3R3,THEM4,AKT3,MAP3K8,ILK,RPS6KB2,CCND1,INPPL1,CDKN1B,MDM2,HSP90AA1,AKT1,TSC2,PDPK1,PIK3R5,MYH4,RPS6KB1,CDC37,PIK3R2,AKT2,INPP5D,CTNNB1,PIK3CB,PIK3CA,EIF4E,GAB1,PIK3R1,CDKN1A,NOS3,RHEB,TSC1	4.639E-4

Table S5. Proteins up- and down-regulated in two tumors and normal colonic mucosas from patients with diabetes. TD means tumors from diabetic patients. T, tumors from non-diabetic patients. ND, normal colonic mucosa from diabetic patients. And N, normal colonic mucosa from non-diabetic patients.

Comparison	Filter (significance)	Up-regulated in condition	Proteins
TDvsT	p-value<0.05	TD	O14602,P04155,P13727,P22090,Q96EY8,Q9H773,Q96C36,O75394,Q9BTM1,P00492,A6NDG6,P55210,Q9H5Q4,Q9NPJ3,Q9Y2S6,O95154,P11908,P00505,P30044,Q9Y5J9,Q9Y3E5,Q9BWD1,Q9Y5L4,O14907,Q9BQC6,Q96EH3,Q01581,Q9H3K6,Q96HS1,Q9BRT2,Q0JRZ9,Q9NRX2,Q969Z0,Q9H0U6,P62328,P99999,Q9Y2Q9,P05141,P20933,P18077,Q9NRN9,A8MWD9,Q96EL3,Q6P161,Q9BYC9,Q9Y2R5,Q13404,Q9UJA5,P49406,P60983,Q86SX6,P61604,P22676,O00182,P62910,Q9H9J2,O14497,P11177,E1BJK2,P15735,Q9C099,P62750,P00491,Q9Y5J7,P53634,Q14376,Q96BR5,Q9H8Y8,Q15185,Q92688,O00584,P62888,P46779,P14406,Q9P0P8,Q8IXM2,Q9Y221,P30405,Q7Z4W1,P49458,Q8NCW5,Q99757,Q9C005,Q8TBX8,P61289,Q01469,Q9NUI1,P62266,P62851,Q9NY12,Q8NFH4,Q9Y5S9,Q9NSA3,P39019,Q8NHP8,Q9BY32,Q8IV56,P02042,O60220,P61088,P56381,Q9NX20,Q9BTZ2,P46776,Q9Y3U8,P47914,P35900,Q9UI30,P37802,O75340,P26373,P49419,P35268,P49207,P50053,Q00688,Q9Y3A6,Q9GZT3,Q9ULC4,P61960,Q16540,P62899,Q8WUH6,Q9Y3B7,Q9BYN8,O60568,Q9Y6H1,Q13907,P62854,P62318,P82664,P49720,P15880,Q96BP2,Q9UKK9,Q9UII2,P63173,P07919,Q9P0M9,Q9Y2S7,Q9NX08,Q8N983,O00154,Q13405,P61313,Q9NZJ9,Q13162,P62277,Q00325,Q9BSH4,Q92598,P16278,O75506,Q9BQ67,P34897,P17931,P62280,Q99519,Q15691,Q13084,Q7Z4G1,Q9Y3D3,Q9BQ61,Q9NY33,P62917,P46781,P32322,P55854,P19387,Q9Y3B4,Q96G21,Q6ZRR9,P42766,Q7L0Y3,P40429,Q92947,P62857,Q96D15,Q5T653,Q6PCE3,O15212,P25788,Q15370,Q96DV4,Q8WXA9,Q92506,P82933,P35222,Q9BRJ2,Q13595,Q9Y399,P61353,Q9H892,P53999,P36551,O75431,Q7Z478,P62877,O43402,Q9NS39,P09211,Q9Y6A4,P04424,P12532,P32969,P62829,P07954,P39023,Q9BZE1,P37837,O14734,P27635,Q02543,P48163,Q9H0A8,P15289,Q9NUL7,Q07021,Q9Y606,Q8TCS8,P52655,P55263,O43169,P18124,P62913,P38117,Q16740,P26639,O14737,Q9BYC8,P62306,O76003,P61970,P62847,Q9H299,Q9H910,P10515,P61326,P10619,P61758,Q9NP97,P48729,Q9NYK5,P37108,P18283,O43324,P05166,P20290,P57105,P49721,Q9NTK5,P20962,Q9NPA8,P04066,O14561,P13798,P78406,P63244,P05386,Q9P015,P62424,Q86WA6,Q15363,P10809,P62906,Q9Y6G5,P27797,Q5EBL8,Q9UMS0,Q9NS69,P62241,Q9NQ50,Q07020,Q9BR76,P01876,Q9NUB1,P61966,Q9UKD2,Q13242,P55145,Q02878,Q9H0N5,P29401,P05455,Q13765,P63220,Q15843,Q9H1E3,Q8IZQ5,P46777,Q9UK76,P62081,P05387,Q9HAB8,P51649,P53597,Q9Y446,P61457,O95336,P35914,P82979,Q5JRX3,Q00059,P63241,Q9P2E9,P83731,P62495,Q9Y3C6,O60869
TDvsT	p-value<0.05	T (down-regulated in TD)	P07996,Q28194_CONT,P02662_CONT,P62834,O14791,P23297,P09493,P16188,Q9H9S4,P01033,P02749,O43294,P52848,P02647,P08493,Q13642,P17661,P22352,O95810,Q3SX28_CONT,Q9Y3S2,Q12888,P01625,P02774,Q9Y3D6,P05362,O15061,Q9NZT1,P51911,O60271,P47895,Q9Y639,Q16853,P14649,P00325,Q99715,P20774,P19823,P00450,Q9BW30,Q8N5H7,P01860,P02768,P52943,P12109,P02765,P53814,P08243,P10909,Q9BZL4,P02766,Q9UMS6,Q9NR12,Q15124,P02748,Q8TAE6,Q53GL0,P29536,P05546,Q9UKA9,P12110,Q5JTV8,Q9UJW0,Q9H4G4,Q14314,P35749,Q9BQE5,Q7Z4I7,P08567,Q63ZY3,Q9Y2W1,P07360,Q5T8P6,P01009,Q04828,Q96JJ7,Q16658,P04258_CONT,Q9UGI8,Q6PCB0,Q96BX8,P21333,Q9BX66,P29590,Q93052,P02747,P02649,P21589,Q9NPQ8,P05090,P00488,P07099,O94811,P09104,P23497,O00159,P02787,Q9Y3B3,P08123,Q14315,O14974,P01008,Q9UI12,O75475,P18206,Q56VL3,O00423,P51608,Q9UBQ7,Q12765,P48739,O95721,Q15628,Q06033,Q9NWV4,P18583
TDvsT	p-value<0.05 + FC>1.2	TD	O14602,P04155,P13727,P22090,Q96EY8,Q9H773,Q96C36,O75394,Q9BTM1,P00492,A6NDG6,P55210,Q9H5Q4,Q9NPJ3,Q9Y2S6,O95154,P11908,P00505,P30044,Q9Y5J9,Q9Y3E5,Q9BWD1,Q9Y5L4,O14907,Q9BQC6,Q96EH3,Q01581,Q9H3K6,Q96HS1,Q9BRT2,Q0JRZ9,Q9NRX2,Q969Z0,Q9H0U6,P62328,P99999,Q9Y2Q9,P05141,P20933,P18077,Q9NRN9,A8MWD9,Q96EL3,Q6P161,Q9BYC9,Q9Y2R5,Q13404,Q9UJA5,P49406,P60983,Q86SX6,P61604,P22676,O00182,P62910,Q9H9J2,O14497,P11177,E1BJK2_CONT,P15735,Q9C099,P62750,P00491,Q9Y5J7,P53634,Q14376,Q96BR5,Q9H8Y8,Q15185,Q92688,O00584,P62888,P46779,P14406,Q9P0P8,Q8IXM2,Q9Y221,P30405,Q7Z4W1,P49458,Q8NCW5,Q99757,Q9C005,Q8TBX8,P61289,Q01469,Q9NUI1,P62266,P62851,Q9NY12,Q8NFH4,Q9Y5S9,Q9NSA3,P39019,Q8NHP8,Q9BY32,Q8IV56,P02042,O60220,P61088,P56381,Q9NX20,Q9BTZ2,P46776,Q9Y3U8,P47914,P35900,Q9UI30,P37802,O75340,P26373,P49419,P35268,P49207,P50053,Q00688,Q9Y3A6,Q9GZT3,Q9ULC4,P61960,Q16540,P62899,Q8WUH6,Q9Y3B7,Q9BYN8,O60568,Q9Y6H1,Q13907,P62854,P62318,P82664,P49720,P15880,Q96BP2,Q9UKK9,Q9UII2,P63173,P07919,Q9P0M9,Q9Y2S7,Q9NX08,Q8N983,O00154,Q13405,P61313,Q9NZJ9,Q13162,P62277,Q00325,Q9BSH4,Q92598,P16278,O75506,Q9BQ67,P34897,P17931,P62280,Q99519,Q15691,Q13084,Q7Z4G1,Q9Y3D3,Q9BQ61,Q9NY33,P62917,P46781,P32322,P55854,P19387,Q9Y3B4,Q96G21,Q6ZRR9,P42766,Q7L0Y3,P40429
TDvsT	p-value<0.05+ FC>1.2	T (down-regulated in TD)	P07996,Q28194_CONT,P02662_CONT,P62834,O14791,P23297,P09493,P16188,Q9H9S4,P01033,P02749,O43294,P52848,P02647,P08493,Q13642,P17661,P22352,O95810,Q3SX28_CONT,Q9Y3S2,Q12888,P01625,P02774,Q9Y3D6,P05362,O15061,Q9NZT1,P51911,O60271,P47895,Q9Y639,Q16853,P14649,P00325,Q99715,P20774,P19823,P00450,Q9BW30,Q8N5H7,P01860,P02768,P52943,P12109,P02765,P53814,P08243,P10909,Q9BZL4,P02766,Q9UMS6,Q9NR12,Q15124,P02748,Q8TAE6,Q53GL0,P29536,P05546,Q9UKA9,P12110,Q5JTV8,Q9UJW0,Q9H4G4,Q14314,P35749,Q9BQE5,Q7Z4I7,P08567,Q63ZY3,Q9Y2W1,P07360,Q5T8P6,P01009,Q04828,Q96JJ7
NDvsN	p-value<0.05	ND	O14602,Q9Y5J9,P01763,P36269,Q9Y6U3,Q9H190,Q9Y2B9,P13761,P62987,O14964,O60218,P12724,A1L188,O14734,Q96EY8,P21741,Q9UBY9,P14854,P16444,Q92506,P42566,Q9UBC5,Q16610,O00748,P30039,O94925,P10109,Q9H6S3,Q16270,P02676,Q9H0U6,Q9Y606,P51970,Q96GE6,Q5JTJ3,Q9UM07,Q96BS2,Q96JY6,Q6UWP2,O75880,Q4U2R6,P02070,P12830,Q96EY5,Q9H3R2,P00403,Q12805,O60220,Q86SZ2,P03950,Q96EL3,P01860,P39060,Q86WA6,Q9Y2S2,P02753,Q9H5X1,Q02978,Q969M7,P46734,P16152,P03928,O95716,Q9UI30,Q96IX5,P08134,Q12864,P62910,Q9BPX5,Q01970,Q08257,P02511,P13611,P18077,O14558,Q9NWU5,O95340,P62873,Q9P2B2,Q9H5N1,Q14197,Q4G0F5
NDvsN	p-value<0.05	N (down-regulated in ND)	P52848,O95678,Q92764,Q9BZL1,O15234,P23297,P15241,P23219,P02652,Q96RU3,Q9NXA8,Q96T23,P02654,Q9Y3D6,Q8TDX6,Q9Y6K5,P02647,Q16795,Q8N5H7,Q9Y244,P33241,P14317,Q63ZY3,O14908,Q14344,P10412,Q6UXN9,Q96JJ3,P00326,P84157,O15247,Q96MU7,P49959,O43813,P20160,P07305,P20774,Q6NXG1,Q9Y3E1,P35269,Q86UX7,P18206,P51608,Q96A08,P50453,Q5SSJ5,Q13303,Q16543,Q13425,P10301,P42226,Q14894,P33992,P52566,P11766,P20962,Q14005,P49915,P33991,Q02790
NDvsN	p-value<0.05 + FC>1.2	ND	O14602,Q9Y5J9,P01763,P36269,Q9Y6U3,Q9H190,Q9Y2B9,P13761,P62987,O14964,O60218,P12724,A1L188,O14734,Q96EY8,P21741,Q9UBY9,P14854,P16444,Q92506,P42566,Q9UBC5,Q16610,O00748,P30039,O94925,P10109,Q9H6S3,Q16270,P02676,Q9H0U6,Q9Y606,P51970,Q96GE6,Q5JTJ3,Q9UM07,Q96BS2,Q96JY6,Q6UWP2,O75880,Q4U2R6,P02070,P12830,Q96EY5,Q9H3R2,P00403,Q12805,O60220,Q86SZ2,P03950,Q96EL3
NDvsN	p-value<0.05+ FC>1.2	N (down-regulated in ND)	P52848,O95678,Q92764,Q9BZL1,O15234,P23297,P15241,P23219,P02652,Q96RU3,Q9NXA8,Q96T23,P02654,Q9Y3D6,Q8TDX6,Q9Y6K5,P02647,Q16795,Q8N5H7,Q9Y244,P33241,P14317,Q63ZY3,O14908,Q14344,P10412
Common proteins in TDvsT and NDvsN	p-value<0.05	TD and ND	O14602,Q9Y5J9,O14734,Q96EY8,Q92506,Q9H0U6,Q9Y606,O60220,Q96EL3,Q86WA6,Q9UI30,P62910,P18077

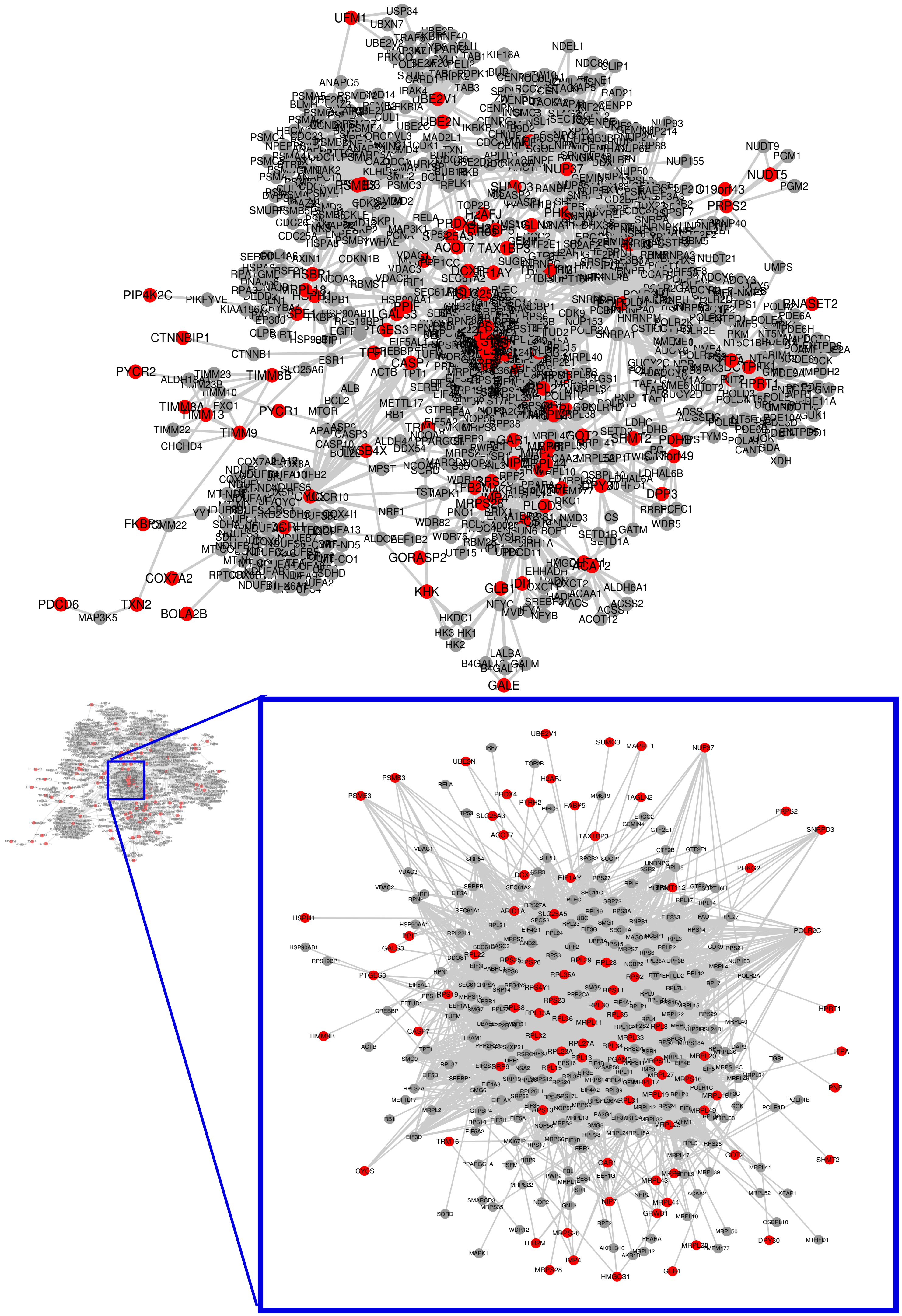

UFM1
USP34
UBXN7
NDEL1
ANAPC5
UBE2V1
UBE2N
NUDT9
PGM1
NUDT5
PGM2
NUP155
NUP37
SUMO3
C19orf43
PRPS2
PIP4K2C
PIKFYVE
CTNNBIP1
CTNNB1
PYCR2
TIMM8B
TIMM8A
TIMM13
PYCR1
TIMM22
TIMM9
CHCHD4
UMPS
RNASET2
COX7A2
PDCD6
MAP3K5
TXN2
BOLA2B
KHK
HKDC1
HK3
HK1
HK2
LALBA
GALM
GALE
DPP3
WDR5
GATM
CS
ALDH6A1
ACSS2
ACOT12
MPST
NRF1
GORASP2
ESR1
SLC25A6
ALB
BCL2
MTOR
SHMT2
XDH
GDA
TYMS
CDKN1B
AXIN1
POLR2C
SNRPD3
HPRT1
ITPA
PNP
GOT2
CYCS
HSPH1
PSME3
PSMB3
MAPRE1
GLB1
HMGCS1
MRPS28
IMP4
TFB2M
DPY30
MTHFD1

Figure S2. Minimal Connected Network of proteins up-regulated in tumors from diabetic patients (TD) compared to tumors from non-diabetic patients (T). In red, differentially expressed proteins with a fold change > 1.2 and a p value <0.05 in diabetic vs non-diabetic conditions.

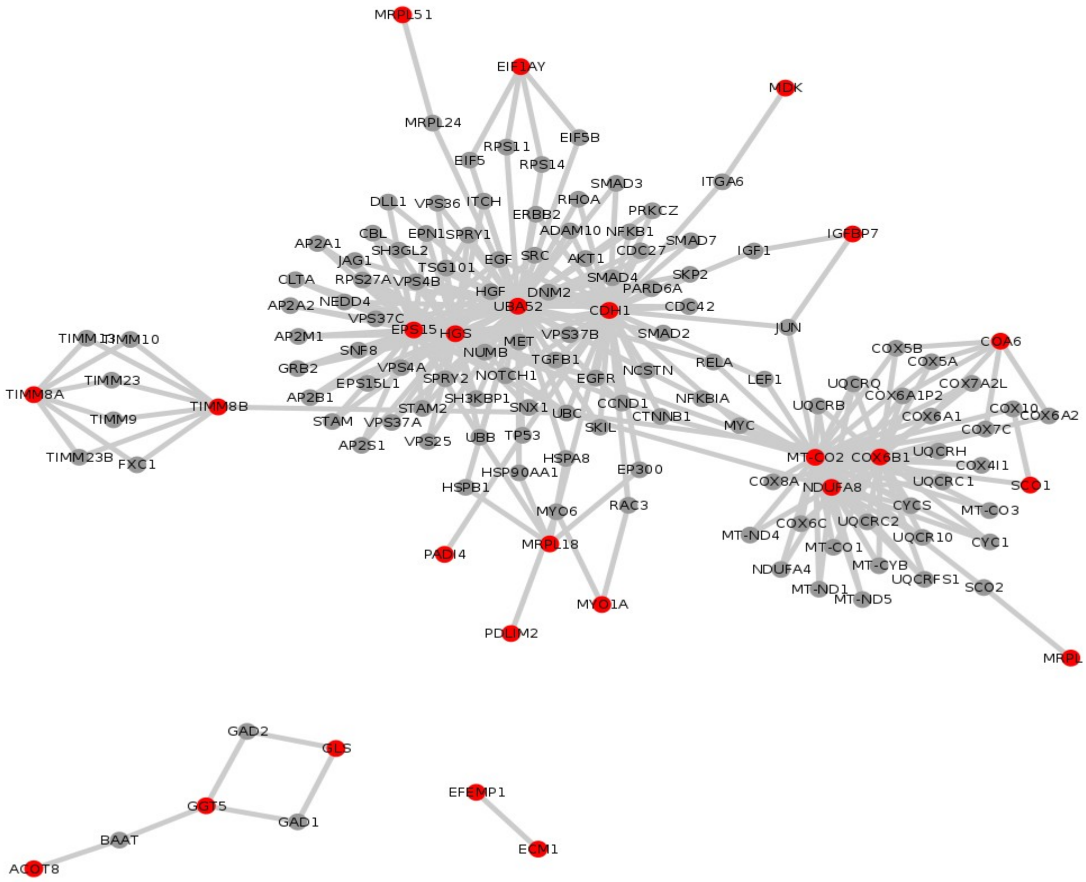

Figure S3. Minimal Connected Network of proteins up-regulated in adjacent mucosa from diabetic patients (ND) compared to adjacent mucosa from non-diabetic patients (N). In red, differentially expressed proteins with a fold change > 1.2 and a p value <0.05 in diabetic vs non-diabetic conditions.

Table S6. Over-represented KEGG pathways in the network made with differentially up-regulated proteins in tumors from diabetic patients compared to tumors from non-diabetic patients.

KEGG id	KEGG pathway name	Annotated proteins in the network	FDR adjusted p-value
hsa00230	Purine metabolism	NME6,POLD3,ADCY2,PDE10A,ADCY3,ADCY5,POLR3A,Q9UKK9,ADCY6,ADCY7,ADCY8,ADCY9,ADSSL1,CANT1,NUDT16,ADSS,DCK,DGUOK,ADCY4,TWISTNB,NT5C2,POLA2,POLR2J2,AMPD1,Q01433,AMPD3,GMPR,GUCY1A2,GUCY1A3,GUCY1B3,GUCY2C,GUCY2F,Q16774,NME7,GUCY2D,NT5C,P50583,P00492,P07741,IMPDH1,P12268,Q9BY32,ENTPD8,ATIC,P15531,P22392,NME3,NME4,P00491,NPR1,NPR2,P21589,AK3,PDE11A,POLR1D,GMPR2,PDE1A,PDE1C,PDE2A,PDE3A,PDE6A,PDE6C,PDE6D,PDE6G,PDE6H,PDE9A,PDE1B,PDE6B,PGM1,P14618,NUDT9,POLE3,POLA1,P28340,P49005,POLE,POLE2,POLR2A,POLR2B,P19387,POLR2D,P19388,POLR2F,POLR2G,P52434,P36954,POLR2J,POLR2K,POLR2L,POLR2J3,Q96G03,PRIM1,POLR3B,ADCY10,P11908,POLE4,NT5M,POLD4,DH,NME5,POLR1B,NT5C1A,PDE5A,Q8TCS8,P49915,ENTPD1,O15160,ENTPD6,ENTPD3,O75356,ENTPD4,GDA,NT5C1B-RDH14,NME1-NME2	1.261E-53
hsa04914	Progesterone-mediated oocyte maturation	P24941,ANAPC10,ADCY2,ADCY3,ADCY5,ADCY6,ADCY7,ADCY8,ADCY9,ADCY4,CDC26,ANAPC2,ANAPC4,P07900,P08238,MAD2L1,FZR1,ANAPC5,ANAPC7,ANAPC11,PLK1,P17612,MAPK1,ANAPC1,BUB1,MAD1L1,CDC23,CDC16,P06493,CDC25A,CDC27	5.995E-6
hsa04932	Non-alcoholic fatty liver disease (NAFLD)	NDUFA11,P13073,COX5B,COX6A1,P14854,COX6C,P14406,P15954,COX8A,CYC1,P05198,UQCRQ,UQCR10,IKBKB,NDUFS7,MAP3K5,MT-CO1,P00403,MT-CO3,NDUFA1,NDUFA2,NDUFA4,NDUFA5,P51970,Q16795,NDUFA10,O14561,NDUFB1,NDUFB2,N	1.894E-20

		DUFB3,NDUFB4,NDUFB5,NDUFB6,NDUFB7,NDUFB8,Q9Y6M9,NDUFB10,NDUFC1,NDUFC2,P28331,O75306,NDUFS3,P49821,O43181,NDUFS5,O75380,NDUFS8,NDUFV2,NDUFV3,NFKB1,Q9P0J0,P99999,Q9NX14,PPARA,NDUFA12,RELA,SDHA,SDHB,SDHC,SDHD,UQCRB,UQCRC1,P22695,UQCRFS1,P07919,P42574,P55210,COX7A2L,P20674,MT-CYB	
hsa05166	HTLV-I infection	CDKN1A,ANAPC10,POLD3,ADCY2,ADCY3,ADCY5,ADCY6,ADCY7,ADCY8,CHUK,ADCY9,CREBBP,P35222,DVL1,DVL2,DVL3,ADCY4,EP300,CDC26,P05141,P12236,ANAPC2,ANAPC4,IKBKB,MAD2L1,MAP3K1,NFKB1,NFKBIA,NFYB,P12004,ANAPC5,ANAPC7,ANAPC11,POLE3,P28340,P49005,POLE,POLE2,P17612,POLE4,POLD4,RB1,CCND1,RELA,BCL2L1,ANAPC1,BUB1B,TP53,P21796,P45880,Q9Y277,XPO1,CDC23,CDC16,BUB3,PTTG1,CDC20,CDC27	1.939E-3
hsa05169	Epstein-Barr virus infection	P24941,O00487,CDKN1A,CDKN1B,TAB1,POLR3A,CHUK,CREBBP,EP300,TAB2,POLR2J2,Q00403,GTF2E1,HSPA6,HSPA8,P04792,IKBKB,ENTPD8,NFKB1,NFKBIA,POLR1D,POLR2A,POLR2B,P19387,POLR2D,P19388,POLR2F,POLR2G,P52434,P36954,POLR2J,POLR2K,POLR2L,POLR2J3,P17612,POLR3B,P62191,P35998,P17980,P43686,PSMC5,P62333,Q99460,Q13200,O43242,P55036,P51665,P48556,O00231,PSMD12,Q9UNM6,RB1,BCL2,RELA,SKP2,MAP3K7,TP53,TRAF6,XPO1,YWHAE,NUP214,ENTPD1,O15160,ENTPD3,P06493,Q15008	9.47E-11
hsa05215	Prostate cancer	P24941,CDKN1A,CDKN1B,CHUK,CREBBP,P35222,EGFR,EP300,MTOR,P07900,P08238,IKBKB,NFKB1,NFKBIA,PDPK1,MAPK1,RB1,CCND1,BCL2,RELA,TP53,HSP90B1,CASP9	2.624E-2
hsa03013	RNA transport	SNUPN,Q86V81,SRRM1,RPP38,NUP50,RNPS1,NUPL2,DDX20,Q8NFH5,EEF1A1,P47813,P05198,P41091,P60842,EIF4A2,P23588,P06730,Q04637,EIF5,O15234,P52298,NUP205,NUP210,NUP160,NUP188,NUP62,Q8TEQ6,UPF2,P11940,NUP43,P60228,P61326,NCBP1,NUP88,GEMIN4,NMD3,PHAX,NUP54,NUP133,NUP107,RANBP2,P46060,UPF1,SEC13,UPF3B,UPF3A,P55854,P12270,XPO1,Q8NFH4,GEMIN7,GEMIN6,NUP85,NUP214,AAAS,P78406,GEMIN2,EIF3A,P55884,Q99613,O15371,O00303,O75821,O15372,Q13347,O75822,P20042,O14602,NUP155,EIF5B,TGS1,NUP93,P38919,NUP153	2.682E-19
hsa05010	Alzheimer's disease	NDUFA11,P13073,COX5B,COX6A1,P14854,COX6C,P14406,P15954,COX8A,CYC1,UQCRQ,UQCR10,APAF1,NDUFS7,MT-CO1,P00403,MT-CO3,NDUFA1,NDUFA2,NDUFA4,NDUFA5,P51970,Q16795,NDUFA10,O14561,NDUFB1,NDUFB2,NDUFB3,NDUFB4,NDUFB5,NDUFB6,NDUFB7,NDUFB8,Q9Y6M9,NDUFB10,NDUFC1,NDUFC2,P28331,O75306,NDUFS3,P49821,O43181,NDUFS5,O75380,NDUFS8,NDUFV2,NDUFV3,Q9P0J0,P99999,Q9NX14,MAPK1,NDUFA12,SDHA,SDHB,SDHC,SDHD,UQCRB,UQCRC1,P22695,UQCRFS1,P07919,P42574,P55210,CASP9,COX7A2L,P20674,MT-CYB	2.186E-15
hsa00240	Pyrimidine metabolism	NME6,POLD3,POLR3A,CANT1,Q5EBM0,UPRT,P17812,DCK,DCTD,TWISTNB,NT5C2,POLA2,POLR2J2,NME7,NT5C,P50583,Q9BY32,ENTPD8,P15531,P22392,NME3,NME4,P00491,P21589,POLR1D,CMPK1,POLE3,POLA1,P28340,P49005,POLE,POLE2,POLR2A,POLR2B,P19387,POLR2D,P19388,POLR2F,POLR2G,P52434,P36954,POLR2J,POLR2K,POLR2L,POLR2J3,PRIM1,POLR3B,CTPS2,POLE4,NT5M,POLD4,TK1,TYMS,P11172,Q9H773,NME5,POLR1B,NT5C1A,Q8TCS8,ENTPD1,O15160,ENTPD6,ENTPD3,O75356,ENTPD4,NT5C1B-RDH14,NME1-NME2	1.308E-30
hsa03015	mRNA surveillance pathway	Q86V81,SRRM1,PAPOLA,RNPS1,CLP1,O43809,Q05048,P33240,CSTF3,P62495,O15234,P52298,SMG1,SMG6,SMG5,UPF2,P11940,P61326,NCBP1,PCF11,CPSF3,CPSF2,P36873,PPP2CA,P30153,PPP2R2A,UPF1,UPF3B,UPF3A,Q8N684,Q6UXN9,Q86U42,P38919,SMG7	4.426E-7
hsa03010	Ribosome	P62854,Q13084,P09001,P42766,MRPL10,RPS4Y2,RPL10L,Q6P5R6,FAU,P40429,Q9Y3U8,Q9BYD1,Q9H0U6,Q9P015,Q9NWU5,RPSA,P62906,Q9Y3D3,MRPS18C,Q71UM5,Q5T653,Q9BYD3,MRPS7,Q9Y399,RPL26L1,RSL24D1,Q9P0M9,Q9NX20,Q9BYC9,Q9NVS2,P82664,P39023,RPL3L,P36578,P46777,Q02878,P18124,P62424,P62917,P32969,P27635,P62913,RPL12,P26373,P61313,P18621,Q07020,Q02543,P84098,RPL21,P35268,P62750,Q16540,P83731,RPL26,P61353,P62888,P46776,P46779,P47914,P62899,P62910,P49207,P18077,RPL36AL,P61927,P61513,P63173,RPL39,RPL41,P83881,P05388,P05386,P05387,P52815,MRPS12,P15880,P23396,RPS3A,RPS4X,P22090,P46782,P62753,P62081,P62241,P46783,P62280,P25398,P62277,P62263,P62841,P62244,P62249,RPS17,P39019,P60866,P63220,P62266,P62847,P62851,RPS27,RPS27A,P62857,RPS29,Q9NRX2,O60783,MRPL14,MRPS15,MRPS11,P82933,P82932,MRPS5,MRPL36,MRPL34,Q9BYC8,Q9Y3B7,MRPL9,MRPL1,P62987,MRPL24,P50914,P62829,O75394,P49406	7.469E-95
hsa00190	Oxidative phosphorylation	NDUFA11,P13073,COX5B,COX6A1,P14854,COX6C,P14406,P15954,COX8A,CYC1,UQCRQ,UQCR10,NDUFS7,P00403,NDUFA1,NDUFA2,NDUFA4,NDUFA5,P51970,Q16795,NDUFA10,O14561,NDUFB1,NDUFB2,NDUFB3,NDUFB4,NDUFB5,NDUFB6,NDUFB7,NDUFB8,Q9Y6M9,NDUFB10,NDUFC1,NDUFC2,P28331,O75306,NDUFS3,P49821,O43181,NDUFS5,O75380,NDUFS8,NDUFV2,NDUFV3,Q9P0J0,Q9NX14,NDUFA12,SDHA,SDHB,SDHC,SDHD,UQCRB,UQCRC1,P22695,UQCRFS1,P07919,COX7A2L,P20674,MT-CO3,MT-CYB,MT-ND1,MT-ND2,MT-ND3,MT-ND4,MT-ND4L,MT-ND5,MT-ND6,MT-CO1	6.797E-23
hsa05012	Parkinson's disease	P30405,ADCY5,NDUFA11,P13073,COX5B,COX6A1,P14854,COX6C,P14406,P15954,COX8A,CYC1,UQCRQ,P05141,P12236,UQCR10,APAF1,NDUFS7,NDUFA1,NDUFA2,NDUFA4,NDUFA5,P51970,Q16795,NDUFA10,O14561,NDUFB1,NDUFB2,NDUFB3,NDUFB4,NDUFB5,NDUFB6,NDUFB7,NDUFB8,Q9Y6M9,NDUFB10,NDUFC1,NDUFC2,P28331,O75306,NDUFS3,P49821,O43181,NDUFS5,O75380,NDUFS8,NDUFV2,NDUFV3,PARK2,Q9P0J0,P99999,Q9NX14,P17612,NDUFA12,SDHA,SDHB,SDHC,SDHD,UBB,UQCRB,UQCRC1,P22695,UQCRFS1,P07919,P21796,P45880,Q9Y277,P42574,CASP9,COX7A2L,P20674,MT-CO1,P00403,MT-CO3,MT-CYB,MT-ND1,MT-ND2,MT-ND3,MT-ND4,MT-ND4L,MT-ND5,MT-ND6	3.311E-32
hsa05016	Huntington's disease	P30405,PPARGC1A,NDUFA11,P13073,COX5B,COX6A1,P14854,COX6C,P14406,P15954,COX8A,CREBBP,CYC1,EP300,POLR2J2,UQCRQ,P05141,P12236,UQCR10,APAF1,NDUFS7,P00403,NDUFA1,NDUFA2,NDUFA4,NDUFA5,P51970,Q16795,NDUFA10,O14561,NDUFB1,NDUFB2,NDUFB3,NDUFB4,NDUFB5,NDUFB6,NDUFB7,NDUFB8,Q9Y6M9,NDUFB10,NDUFC1,NDUFC2,P28331,O75306,NDUFS3,P49821,O43181,NDUFS5,O75380,NDUFS8,NDUFV2,NDUFV3,NRF1,Q9P0J0,P99999,POLR2A,POLR2B,P19387,POLR2D,P19388,POLR2F,POLR2G,P52434,P36954,POLR2J,POLR2K,POLR2L,Q9NX14,POLR2J3,NDUFA12,SDHA,SDHB,SDHC,SDHD,TAF4,TP53,UQCRB,UQCRC1,P22695,UQCRFS1,P07919,P21796,P45880,Q9Y277,P42574,CASP9,COX7A2L,P20674,MT-CYB,MT-CO3, MT-CO1	2.586E-27
hsa03040	Spliceosome	Q86V81,Q01081,Q15459,PRPF8,TXNL4A,Q12874,Q13435,P26368,HNRNPA3,P52298,O75643,Q15393,SF3B1,PRPF6,P38159,P09651,P07910,P61978,HNRNPU,HSPA6,HSPA8,P61326,HNRNPM,N	1.320E-12

		CBP1,Q15365,CDC40,Q07955,SRSF2,P84103,Q08170,Q13243,Q13247,Q16629,P08621,P09012,P09661,SNRPB,P08579,SNRPD1,P62316,P62318,SNRPE,P62306,SNRPG,Q15428,Q9BWJ5,Q7RTV0,Q13242,O43172,Q15029,Q96DI7,DDX23,P38919,DHX38	
hsa04120	Ubiquitin mediated proteolysis	Q9UNE7,ANAPC10,UBE2C,CDC26,ANAPC2,ANAPC4,MAP3K1,PARK2,FZR1,ANAPC5,ANAPC7,ANAPC11,SMURF1,RFWD2,ANAPC1,P63208,SKP2,TRAF6,UBE2A,UBE2B,UBE2D1,UBE2D2,UBE2E1,P61088,CUL3,Q13616,CDC23,CDC16,BTRC,KEAP1,CDC20,CDC27,P62877	1.322E-2
hsa03020	RNA polymerase	POLR3A,TWISTNB,POLR2J2,POLR1D,POLR2A,POLR2B,P19387,POLR2D,P19388,POLR2F,POLR2G,P52434,P36954,POLR2J,POLR2K,POLR2L,POLR2J3,POLR3B,POLR1B,O15160	3.218E-9
hsa04110	Cell cycle	P24941,CDKN1A,CDKN1B,STAG1,ANAPC10,STAG2,CREBBP,EP300,CDC26,ANAPC2,ANAPC4,MAD2L1,ORC1,P12004,FZR1,ANAPC5,ANAPC7,ANAPC11,PLK1,RAD21,RB1,CCND1,ANAPC1,P63208,SKP2,BUB1,BUB1B,TP53,YWHAE,MAD1L1,Q13616,CDC23,CDC16,CCNH,SMC3,BUB3,PTTG1,ESPL1,P06493,CDC6,CDC20,CDC25A,CDC27,P62877	2.964E-8
hsa04114	Oocyte meiosis	P24941,ANAPC10,ADCY2,ADCY3,ADCY5,ADCY6,ADCY7,ADCY8,ADCY9,SGOL1,ADCY4,CDC26,ANAPC2,ANAPC4,MAD2L1,ANAPC5,ANAPC7,ANAPC11,PLK1,P36873,PPP2CA,P30153,P17612,MAPK1,ANAPC1,P63208,AURKA,BUB1,YWHAE,Q13616,CDC23,CDC16,BTRC,SMC3,PTTG1,ESPL1,P06493,CDC20,CDC27,P62877	1.258E-7
hsa03008	Ribosome biogenesis in eukaryotes	RCL1,O00567,RPP38,WDR36,O60832,P22087,GTPBP4,GNL3,P56537,NMD3,NOP58,Q9NY12,GNL3L,RBM28,Q9NV31,Q9NX24,UTP6,PWP2,XPO1,EFTUD1,WDR75,UTP15,CIRH1A,Q96G21	2.255E-3
hsa03050	Proteasome	P61289,O00487,PSMB11,PSMA8,PSME4,PSMA1,P25788,P25789,P28066,P60900,O14818,PSMB1,P49721,P49720,PSMB4,PSMB5,PSMB6,PSMB7,P40306,P62191,P35998,P17980,P43686,PSMC5,P62333,Q99460,Q13200,O43242,P55036,P51665,P48556,O00231,PSMD12,Q9UNM6,Q06323,Q9UL46,Q92530,Q15008	4.002E-25
hsa03022	Basal transcription factors	ERCC2,ERCC3,P52655,Q00403,GTF2E1,P35269,P13984,GTF2H1,GTF2H3,MNAT1,TAF1,TAF4,TAF6,CCNH	3.607E-2
hsa03420	Nucleotide excision repair	POLD3,ERCC2,ERCC3,GTF2H1,GTF2H3,MNAT1,P12004,POLE3,P28340,P49005,POLE,POLE2,POLE4,POLD4,P27694,RPA2,RPA3,CCNH,P62877	8.414E-5
hsa00010	Glycolysis / Gluconeogenesis	GALM,LDHAL6A,ALDOA,GCK,HK1,HK2,P52790,LDHB,LDHC,P49419,P11177,PGM1,P14618,Q96G03,ACSS2,HKDC1,Q9NUB1,LDHAL6B	4.494E-2
hsa04260	Cardiac muscle contraction	P13073,COX5B,COX6A1,P14854,COX6C,P14406,P15954,COX8A,CYC1,UQCRQ,UQCR10,MT-CO1,P00403,MT-CO3,MT-CYB,UQCRB,UQCRC1,P22695,UQCRFS1,P07919,COX7A2L,P20674	1.017E-2
hsa03030	DNA replication	POLD3,POLA2,P12004,POLE3,POLA1,P28340,P49005,POLE,POLE2,PRIM1,POLE4,POLD4,P27694,RPA2,RPA3	6.906E-4
hsa03060	Protein export	SEC61B,SEC11A,SEC61G,SPCS1,SEC61A1,SEC61A2,SRPRB,SPCS3,P49458,P37108,SRP19,P61011,SRP68,SRP72,P08240,SEC11C,SPCS2	3.139E-9
hsa00052	Galactose metabolism	GALM,P15121,Q14376,GCK,B4GALT1,P16278,HK1,HK2,P52790,LALBA,PGM1,Q96G03,O60218,HKDC1,B4GALT2	4.884E-5
hsa00524	Butirosin and neomycin biosynthesis	GCK,HK1,HK2,P52790,HKDC1	7.605E-4

Table S7. Over-represented KEGG pathways in the network made with differentially up-regulated proteins in normal colonic mucosa from diabetic patients compared to normal colonic mucosa from non-diabetic patients.

KEGG id	KEGG pathway name	Annotated proteins in the network	FDR adjusted p-value
hsa04010	MAPK signaling pathway	EGF,EGFR,AKT1,GRB2,HSPA8,P04792,JUN,MYC,NFKB1,P60763,RELA,TGFB1,TP53,CDC42	4.310E-3
hsa04012	ErbB signaling pathway	EGF,EGFR,ERBB2,AKT1,GRB2,JUN,MYC,SRC,CBL	6.139E-4
hsa04014	Ras signaling pathway	EGF,EGFR,AKT1,GRB2,HGF,IGF1,RHOA,MET,NFKB1,P60763,RELA,CDC42	1.261E-2
hsa04015	Rap1 signaling pathway	P35222,EGF,EGFR,AKT1,HGF,IGF1,RHOA,MET,PARD6A,PRKCZ,P60763,SRC,CDC42,P12830	9.278E-4
hsa04066	HIF-1 signaling pathway	EGF,EGFR,EP300,ERBB2,AKT1,IGF1,NFKB1,RELA	7.196E-3
hsa04068	FoxO signaling pathway	EGF,EGFR,EP300,AKT1,GRB2,IGF1,SMAD2,SMAD3,SMAD4,CCND1,SKP2,TGFB1	1.985E-4
hsa04151	PI3K-Akt signaling pathway	EGF,EGFR,AKT1,GRB2,HGF,P07900,IGF1,ITGA6,MET,MYC,NFKB1,CCND1,RELA,TP53	4.440E-2
hsa04210	Apoptosis	AKT1,NFKB1,NFKBIA,P99999,RELA,TP53	3.683E-2
hsa04370	VEGF signaling pathway	AKT1,P04792,P60763,SRC,CDC42	3.615E-2
hsa04510	Focal adhesion	P35222,EGF,EGFR,ERBB2,AKT1,GRB2,HGF,IGF1,ITGA6,JUN,RHOA,MET,P60763,CCND1,SRC,CDC42	5.072E-5
hsa04550	Signaling pathways regulating pluripotency of stem cells	P35222,AKT1,GRB2,IGF1,SMAD2,SMAD3,SMAD4,MYC,SKIL	1.355E-2

hsa04660	T cell receptor signaling pathway	AKT1,GRB2,JUN,RHOA,NFKB1,NFKBIA,RELA,CBL,CDC42	2.E-3
hsa04662	B cell receptor signaling pathway	AKT1,GRB2,JUN,NFKB1,NFKBIA,P60763,RELA	4.606E-3
hsa04668	TNF signaling pathway	JAG1,AKT1,JUN,NFKB1,NFKBIA,RELA,ITCH	3.535E-2
hsa04722	Neurotrophin signaling pathway	AKT1,GRB2,JUN,RHOA,NFKB1,NFKBIA,RELA,TP53,CDC42	4.774E-3
hsa04915	Estrogen signaling pathway	EGFR,AKT1,GRB2,HSPA8,P07900,JUN,SRC	2.094E-2
hsa04917	Prolactin signaling pathway	AKT1,GRB2,NFKB1,CCND1,RELA,SRC	1.807E-2
hsa04919	Thyroid hormone signaling pathway	P35222,EP300,AKT1,MYC,NOTCH1,CCND1,SRC,TP53	1.496E-2
hsa04932	Non-alcoholic fatty liver disease (NAFLD)	P13073,COX5B,COX6A1,COX6A2,P14854,COX6C,P15954,COX8A,CYC1,AKT1,UQCRQ,UQCR10,JUN,MT-CO1,P00403,MT-CO3,MT-CYB,NDUFA4,P51970,NFKB1,P99999,RELA,TGFB1,UQCRB,UQCRC1,P22695,UQCRFS1,P07919,ITCH,COX7A2L,P20674,CDC42	2.698E-22
hsa04933	AGE-RAGE signaling pathway in diabetic complications	AKT1,JUN,SMAD2,SMAD3,SMAD4,NFKB1,PRKCZ,CCND1,RELA,TGFB1,CDC42	9.078E-5
hsa05142	Chagas disease (American trypanosomiasis)	AKT1,JUN,SMAD2,SMAD3,NFKB1,NFKBIA,RELA,TGFB1	7.507E-3
hsa05145	Toxoplasmosis	AKT1,HSPA8,ITGA6,NFKB1,NFKBIA,P99999,RELA,TGFB1	1.496E-2
hsa05160	Hepatitis C	EGF,EGFR,AKT1,GRB2,NFKB1,NFKBIA,RELA,TP53	2.894E-2
hsa05161	Hepatitis B	EP300,AKT1,GRB2,JUN,SMAD3,SMAD4,MYC,NFKB1,NFKBIA,P99999,CCND1,RELA,SRC,TGFB1,TP53	4.572E-6
hsa05166	HTLV-I infection	P35222,EP300,AKT1,JUN,SMAD2,SMAD3,SMAD4,MYC,NFKB1,NFKBIA,CCND1,RELA,TGFB1,TP53,CDC27	1.867E-3
hsa05169	Epstein-Barr virus infection	EP300,AKT1,HSPA8,P04792,JUN,MYC,NEDD4,NFKB1,NFKBIA,RELA,SKP2,TP53	4.774E-3
hsa05200	Pathways in cancer	P35222,EGF,EGFR,EP300,ERBB2,AKT1,GRB2,HGF,P07900,IGF1,ITGA6,JUN,RHOA,SMAD2,SMAD3,SMAD4,MET,MYC,NFKB1,NFKBIA,LEF1,P99999,P60763,CCND1,RELA,SKP2,TGFB1,TP53,CBL,CDC42,P12830	1.413E-9
hsa05205	Proteoglycans in cancer	P35222,EGFR,ERBB2,AKT1,GRB2,HGF,IGF1,RHOA,SMAD2,MET,MYC,CCND1,SRC,TGFB1,TP53,CBL,CDC42	1.250E-5
hsa05210	Colorectal cancer	P35222,AKT1,JUN,RHOA,SMAD2,SMAD3,SMAD4,MYC,LEF1,P99999,P60763,CCND1,TGFB1,TP53	5.385E-10
hsa05211	Renal cell carcinoma	EP300,AKT1,GRB2,HGF,JUN,MET,TGFB1,CDC42	5.616E-4
hsa05212	Pancreatic cancer	EGF,EGFR,ERBB2,AKT1,SMAD2,SMAD3,SMAD4,NFKB1,P60763,CCND1,RELA,TGFB1,TP53,CDC42	1.188E-9
hsa05213	Endometrial cancer	P35222,EGF,EGFR,ERBB2,AKT1,GRB2,MYC,LEF1,CCND1,TP53,P12830	1.184E-7
hsa05214	Glioma	EGF,EGFR,AKT1,GRB2,IGF1,CCND1,TP53	2.418E-3
hsa05215	Prostate cancer	P35222,EGF,EGFR,EP300,ERBB2,AKT1,GRB2,P07900,IGF1,NFKB1,NFKBIA,LEF1,CCND1,RELA,TP53	5.542E-9
hsa05218	Melanoma	EGF,EGFR,AKT1,HGF,IGF1,MET,CCND1,TP53,P12830	1.656E-4
hsa05220	Chronic myeloid leukemia	AKT1,GRB2,SMAD3,SMAD4,MYC,NFKB1,NFKBIA,CCND1,RELA,TGFB1,TP53,CBL	4.452E-7
hsa05221	Acute myeloid leukemia	AKT1,GRB2,MYC,NFKB1,LEF1,CCND1,RELA	1.232E-3
hsa05222	Small cell lung cancer	AKT1,ITGA6,MYC,NFKB1,NFKBIA,P99999,CCND1,RELA,SKP2,TP53	1.232E-4
hsa05223	Non-small cell lung cancer	EGF,EGFR,ERBB2,AKT1,GRB2,CCND1,TP53	1.132E-3
hsa05230	Central carbon metabolism in cancer	EGFR,ERBB2,AKT1,O94925,MET,MYC,TP53,O43819	6.052E-4
hsa05203	Viral carcinogenesis	EP300,GRB2,JUN,RHOA,NFKB1,NFKBIA,CCND1,RELA,SKP2,SRC,TP53,CDC42	5.981E-3
hsa04144	Endocytosis	STAM2,SNF8,Q96CW1,AP2S1,P09496,Q8NEZ2,AP2A1,AP2A2,P63010,P50570,EGF,EGFR,P42566,Q9UN37,Q9Y6I3,Q96B97,HSPA8,RHOA,SMAD2,SMAD3,MET,NEDD4,PARD6A,VPS36,VPS37C,PRKCZ,EPS15L1,SH3GL2,SNX1,SRC,TGFB1,TSG101,VPS37B,STAM,ITCH,VPS25,CBL,O14964,O75351,CDC42	9.332E-23

hsa05206	MicroRNAs in cancer	SPRY2,EGFR,EP300,ERBB2,O94925,GRB2,RHOA,MET,MYC,NFKB1,NOTCH1,CCND1,TP53	1.261E-2
hsa04310	Wnt signaling pathway	P35222,EP300,JUN,RHOA,SMAD3,SMAD4,MYC,LEF1,P60763,CCND1,TP53	1.130E-3
hsa05010	Alzheimer's disease	ADAM10,P13073,COX5B,COX6A1,COX6A2,P14854,COX6C,P15954,COX8A,CYC1,NCSTN,UQCRQ,UQCR10,MT-CO1,P00403,MT-CO3,MT-CYB,NDUFA4,P51970,P99999,UQCRB,UQCRC1,P22695,UQCRFS1,P07919,COX7A2L,P20674	9.090E-16
hsa00190	Oxidative phosphorylation	P13073,COX5B,COX6A1,COX6A2,P14854,COX6C,P15954,COX8A,COX10,CYC1,UQCRQ,UQCR10,MT-CO1,P00403,MT-CO3,MT-CYB,MT-ND1,MT-ND4,MT-ND5,NDUFA4,P51970,UQCRB,UQCRC1,P22695,UQCRFS1,P07919,COX7A2L,P20674	1.419E-19
hsa05012	Parkinson's disease	P13073,COX5B,COX6A1,COX6A2,P14854,COX6C,P15954,COX8A,CYC1,UQCRQ,UQCR10,MT-CO1,P00403,MT-CO3,MT-CYB,MT-ND1,MT-ND4,MT-ND5,NDUFA4,P51970,P99999,UBB,UQCRB,UQCRC1,P22695,UQCRFS1,P07919,COX7A2L,P20674	9.7E-20
hsa05016	Huntington's disease	Q96CW1,AP2S1,P09496,P13073,COX5B,COX6A1,COX6A2,P14854,COX6C,P15954,COX8A,CYC1,AP2A1,AP2A2,P63010,EP300,UQCRQ,UQCR10,NDUFA4,P51970,P99999,TP53,UQCRB,UQCRC1,P22695,UQCRFS1,P07919,COX7A2L,P20674,MT-CYB,MT-CO1,P00403,MT-CO3	4.338E-20
hsa04350	TGF-beta signaling pathway	EP300,RHOA,SMAD2,SMAD3,SMAD4,SMAD7,MYC,TGFB1	2.191E-3
hsa05100	Bacterial invasion of epithelial cells	P09496,P35222,P50570,RHOA,MET,SRC,CBL,CDC42,P12830	3.162E-4
hsa05131	Shigellosis	NFKB1,NFKBIA,RELA,SRC,CDC42	4.806E-2
hsa04520	Adherens junction	P35222,EGFR,EP300,ERBB2,RHOA,SMAD2,SMAD3,SMAD4,MET,LEF1,P60763,SRC,CDC42,P12830	4.112E-9
hsa04110	Cell cycle	EP300,SMAD2,SMAD3,SMAD4,MYC,CCND1,SKP2,TGFB1,TP53,CDC27	1.713E-3
hsa05219	Bladder cancer	EGF,EGFR,ERBB2,MYC,CCND1,SRC,TP53,P12830	2.365E-5
hsa05120	Epithelial cell signaling in Helicobacter pylori infection	ADAM10,EGFR,JUN,MET,NFKB1,NFKBIA,RELA,SRC,CDC42	1.232E-4
hsa04390	Hippo signaling pathway	P35222,SMAD2,SMAD3,SMAD4,SMAD7,MYC,PARD6A,LEF1,PRKCZ,CCND1,TGFB1,P12830	6.139E-4
hsa04721	Synaptic vesicle cycle	Q96CW1,AP2S1,P09496,AP2A1,AP2A2,P63010,P50570	2.090E-3
hsa05216	Thyroid cancer	P35222,MYC,LEF1,CCND1,TP53,P12830	2.630E-4
hsa00430	Taurine and hypotaurine metabolism	GAD1,GAD2,P36269,BAAT	5.474E-4
hsa04260	Cardiac muscle contraction	P13073,COX5B,COX6A1,COX6A2,P14854,COX6C,P15954,COX8A,CYC1,UQCRQ,UQCR10,MT-CO1,P00403,MT-CO3,MT-CYB,UQCRB,UQCRC1,P22695,UQCRFS1,P07919,COX7A2L,P20674	3.358E-18
hsa04330	Notch signaling pathway	JAG1,EP300,NCSTN,DLL1,NOTCH1,NUMB	2.823E-3
hsa05134	Legionellosis	HSPA8,NFKB1,NFKBIA,P99999,RELA	2.6263E-2
hsa04961	Endocrine and other factor-regulated calcium reabsorption	Q96CW1,AP2S1,P09496,AP2A1,AP2A2,P63010,P50570	4.678E-4
hsa05321	Inflammatory bowel disease (IBD)	JUN,SMAD2,SMAD3,NFKB1,RELA,TGFB1	1.202E-2

Table S8. Over-represented KEGG pathways in tumors from diabetic patients (TD) and normal colonic mucosas from diabetic patients (ND) compared to tumors from non-diabetic patients (T) and normal colonic mucosas from non-diabetic patients (N) respectively.

KEGG id	KEGG pathway name
hsa04932	Non-alcoholic fatty liver disease (NAFLD)
hsa05166	HTLV-I infection
hsa05169	Epstein-Barr virus infection
hsa05215	Prostate cancer
hsa05010	Alzheimer's disease
hsa00190	Oxidative phosphorylation
hsa05012	Parkinson's disease
hsa05016	Huntington's disease

hsa04110	Cell cycle
hsa04260	Cardiac muscle contraction

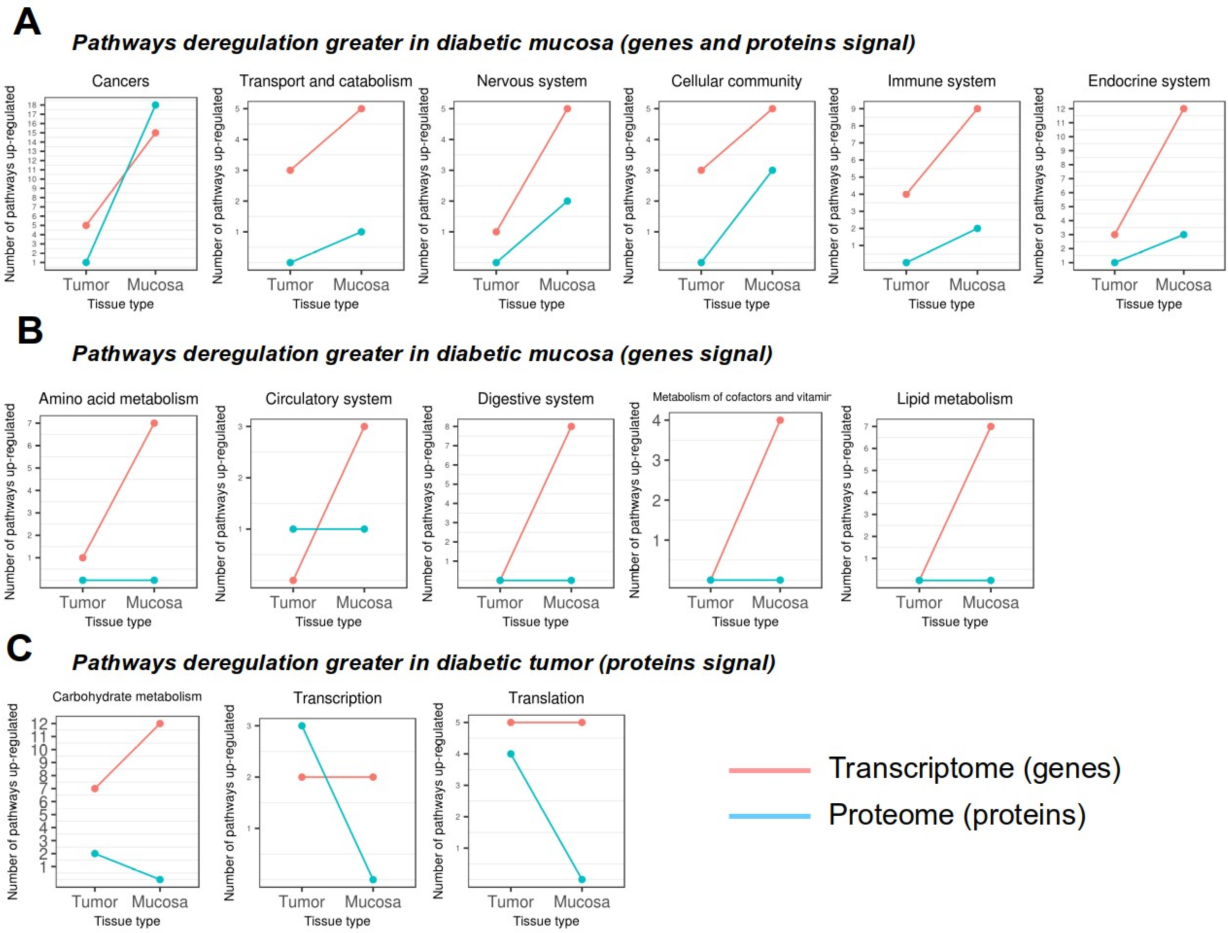

Figure S4. (**A,B,C**) General trends in deregulation of genes and proteins in diabetic tumor and diabetic mucosa. We calculated the number of over-represented pathways (classified by super-classes) in the two initial comparisons (diabetic tumor versus non-diabetic tumor and diabetic normal colonic mucosa versus non-diabetic normal colonic mucosa), then we compare these number from in each tissue (diabetic tumor or diabetic mucosa) to extract general trends in the deregulation of pathways in tumors and mucosa under the effect of diabetes.
